# Evaluation of newly synthesized schiff base Pd(II) complexes for prostate cancer treatment through *in vitro* cytotoxicity and molecular mechanistic studies

**DOI:** 10.3389/fchem.2025.1636477

**Published:** 2025-07-17

**Authors:** Damnjan Pantic, Nikola Mirkovic, Tatjana Vulovic, Danijela Jovanovic, Stefan Jakovljevic, Petar Canovic, Milan Zaric, Radica Zivkovic Zaric, Marina Kostic, Jovana Dragojevic, Vera Divac, Ziko Milanovic, Kristina Milisavljevic, Marina Mitrovic, Ivanka Zelen

**Affiliations:** ^1^ Department of Surgery, Faculty of Medical Sciences, University of Kragujevac, Kragujevac, Serbia; ^2^ Department of Urology, Clinic of Urology and Nephrology, University Clinical Center Kragujevac, Kragujevac, Serbia; ^3^ Department of Vascular Surgery Center, University Clinical Center Kragujevac, Kragujevac, Serbia; ^4^ Department of Anesthesia and Resuscitation, University Clinical Center Kragujevac, Kragujevac, Serbia; ^5^ Department of General Surgery, University Clinical Center Kragujevac, Kragujevac, Serbia; ^6^ Department of Biochemistry, Faculty of Medical Sciences, University of Kragujevac, Kragujevac, Serbia; ^7^ Department of Pharmacology and Toxicology, Faculty of Medical Sciences, University of Kragujevac, Kragujevac, Serbia; ^8^ Department of Clinical Pharmacology, University Clinical Center Kragujevac, Kragujevac, Serbia; ^9^ Department of Science, Institute for Information Technologies, University of Kragujevac, Kragujevac, Serbia; ^10^ Department of Chemistry, Faculty of Sciences, University of Kragujevac, Kragujevac, Serbia

**Keywords:** palladium, schiff bases, DNA, albumin, cytotoxicity, apoptosis, prostate cancer

## Abstract

**Introduction:**

Palladium (II) complexes are promising anticancer agents with potential advantages over platinum drugs. This study aimed to synthesize and characterize three new Pd(II) complexes (**2a–2c**) with Schiff base ligands derived from salicylic acid and amine scaffolds, and to evaluate their antitumor activity against prostate cancer cells.

**Methods:**

The Pd(II) complexes were synthesized and structurally characterized. Cytotoxicity was tested on two human prostate cancer cell lines (PC-3, DU-145) and healthy fibroblasts (MRC-5). Apoptosis induction was assessed by flow cytometry, with a focus on Bcl-2 and caspase proteins. Molecular docking was used to examine binding to the androgen receptor (AR) and apoptotic regulators (CASP3, BCL2, BAX). DNA and human serum albumin (HSA) binding were also investigated.

**Results:**

All complexes showed significant cytotoxicity. Notably, complex **2c** exhibited more potent cytotoxic activity than cisplatin in prostate cancer cell lines, with lower IC_50_ values after 72 h exposure in DU-145 (7.1 µM vs. 8.2 µM) and PC-3 cells (8.6 µM vs. 21.9 µM), while showing reduced toxicity in normal MRC-5 cells (42.3 µM vs. 24.4 µM). Apoptosis was confirmed as the primary cytotoxic mechanism, involving the activation of Bcl-2 and caspases. Docking studies revealed that complex **2c** had the strongest binding affinity to AR and apoptotic proteins, mediated by hydrogen bonds, π–π stacking, and hydrophobic interactions. DNA and HSA binding supported their biological relevance.

**Conclusion:**

Complex **2c** exhibits potent anticancer activity through the induction of apoptosis and dual targeting of the AR and apoptotic pathways, making it a promising candidate for further development of anticancer drugs.

## 1 Introduction

The treatment of cancer is the most challenging issue of modern science, particularly due to the resistance of tumor cells to many utilized protocols and treatments, as well as due to serious side effects. Metal complexes have emerged as a suitable alternative for the treatment of different ailments due to their unique properties (Makarem et al., 2025; Klika et al., 2022). Among them, platinum-based drugs still represent the most explored options for cancer treatment, but the new studies concentrate on other metal drugs with better efficacy (Gupta et al., 2018; Cross et al., 2016; Galanski et al., 2003; Raza et al., 2021; Prathima et al., 2023). The idea behind the design of new metalotherapeutics with increased activity and lowered toxicity is based mainly on the elucidation of antitumor molecular mechanisms different from those expressed by platinum compounds (Prathima et al., 2023). The last two decades have brought increased interest in the design and synthesis of palladium complexes that could serve as alternative anticancer drugs (Lazarević et al., 2017; Vojtek et al., 2019; Carneiro et al., 2020) inspired by their coordination mode similar to that of platinum, but, according to some studies performed on rats, significantly lower toxicity (Abu-Surrah and Kettunen, 2006). As it undergoes a redox reaction, the palladium is also capable of forming reactive oxygen species (ROS). Despite the obvious similarity of these two metalotherapeutic groups, the palladium complexes are much more reactive, therefore, imposing the need for their stabilization through the utilization of specific chelating ligands. The choice of a suitable ligand is a crucial step in the development of a promising antitumor candidate, knowing that finely tuned physicochemical properties of the used ligand, its biological potential, and the strength of the ligand-metal bond directly influence the selectivity and mechanism of the antitumor activity at a molecular level.

The Schiff bases represent molecules widely utilized in many biological studies as pharmacophores capable of forming stable complexes with a variety of metals. Besides the nitrogen atom from imino groups, the molecular scaffold of Schiff bases can be easily manipulated to contain other donor atoms, such as oxygen and sulfur, and, in that way, provide the desired physicochemical or pharmaceutical properties necessary for the establishment of targeted biological activity. There are numerous reports evidencing the pharmacological potential of these compounds reflected in pronounced antitubercular, anti-inflammatory, antiviral, anticholinesterase, antibacterial, anticancer, and antioxidant activities (Aslan et al., 2020; Hosseini-Yazdi et al., 2017; Gowdhami et al., 2021; Kamel et al., 2010; Liu et al., 2019; Thiriveedhi et al., 2019; Amin et al., 2019; Lahmidi et al., 2019). When it comes to the tumor cells, Schiff bases can be selectively hydrolyzed by tumor cells or provide the release of active amines as antimetabolites (Shi et al., 2020). Other antitumor effects of Schiff bases include the cell cycle arrest by the production of free radicals and damage to proteins and nucleic acids (Panchsheela Ashok et al., 2020; Dhivya et al., 2015; Ma et al., 2012). These multiple biological actions make Schiff bases particularly attractive ligands for the design of metal complexes with enhanced therapeutic profiles. Despite the significant number of studies on Schiff base metal complexes, the exploration of palladium (II) complexes incorporating biogenic amine motifs such as tryptamine and tyramine remains limited, providing an opportunity for the development of new structures with potentially improved selectivity and reduced toxicity. Moreover, the rational inclusion of *p*-hydroxybenzylamine, structurally related to tyramine but with an additional hydroxyl group, could further modulate the physicochemical properties and biological activity of the resulting complexes.

As a consequence of above mentioned, the Schiff bases are also utilized as a ligand for the design of palladium complexes as promising candidates for photodynamic therapy (Demirbağ et al., 2024), as well as effective anticancer agents against a variety of cancer cell lines through the different molecular pathways, such as inhibition of proteasomes, enzymes, and cancer protein markers (Podolski-Renić et al., 2024). There are numerous literature examples describing the potential of Schiff base-derived palladium complexes as promising anticancer agents. For example, palladium complex derived from 2-[({2-[(2-hydroxyethyl)amino]ethyl}imino)methyl]phenol has demonstrated potent anticancer activity against several cancer cell lines, with respective IC_50_ values of 13.24 ± 1.21, 25.24 ± 0.91, 38.14 ± 1.19, and 31.21 ± 2.56 µM against HT-1080, A-549, MCF-7, and MDA-MB-231 (Khaldoune et al., 2023). The Schiff base made from (*S*)-2-amino-3-phenyl-1-propanol with 2-hydroxybenzaldehyde and 2-hydroxy-1-naphthaldehyde has been utilized for the synthesis of a palladium complex that exhibited anticancer activity against DLD-1 and MDA-MB-231 cell lines with IC_50_ values of 4.07 and 9.97 µM, respectively (Basaran et al., 2022). Also, palladium complex made from Schiff base comprising heterocyclic core in the structure was more efficient than cisplatin in killing breast cancer cells with an IC_50_ dose (31.80 ± 4.05 µM) (Al-Azzawi et al., 2025).

Among the cancers, prostate cancer is one of the leading causes of male death. The existing therapeutic strategies, such as androgen deprivation therapy, are associated with increased resistance, and new approaches in the treatment are necessary. One of the current promising ways in this domain is the development of strategies for the effective delivery of miR-34 to prostate cancer cells (Abdelaal et al., 2024). Although these attempts are very often associated with delivery vehicle-associated toxicity and non-adequate stability and cell uptake, one of the possible solutions is an approach based on small molecule ligand conjugation to miR-34 without a delivery vehicle, a way that enables high affinity and specificity for receptors (Abdelaal et al., 2024; Li et al., 2024).

In the light of the foregoing, herein we report the synthesis, characterization, and results of *in vitro* cytotoxic assay of three new palladium (II) complexes bearing Schiff base ligands combining salicylic aldehyde and tryptamine/tyramine and *p*-OH benzylamine structural motifs. The novelty of this study lies in the strategic selection of biologically relevant amine scaffolds and the investigation of their combined effects with palladium (II) coordination on anticancer potential. While tryptamine and tyramine represent examples of biogenic amines, *p*-OH benzylamine was used due to the structural resemblance to the tyramine scaffold. The cytotoxic activity was assessed on two human prostate cancer cell lines, PC-3 and DU-145, and the results were compared with those obtained with the healthy fibroblast cell line MRC-5. It was of interest to compare the influence of the palladium metal ion coordination to the Schiff base ligand on their antitumor potential. To gain further insight into their possible mode of action, we focused on one of the key molecular targets in prostate cancer–the androgen receptor (AR). This receptor plays a central role in disease progression by regulating the transcription of genes involved in cell growth, survival, and differentiation upon binding with androgens such as testosterone and dihydrotestosterone (Chatterjee, 2003). Notably, AR signaling remains active even under androgen-deprived conditions in tumor cells, facilitating continued tumor proliferation and the emergence of therapy resistance (Li et al., 2025). Taking into consideration the strong connection between androgen-AR signaling and prostate cancers, some of the available options for treatments are based on the application of androgen deprivation therapy (ADT) or direct targeting by anti-androgens. Although these approaches result in cell cycle arrest and apoptosis induction, due to the disease progression and drug resistance, new therapeutic strategies are needed (Culig and Puhr, 2024; Fujita and Nonomura, 2019; Mehralivand et al., 2024).

Given its critical role, a molecular docking study was undertaken to evaluate the interaction of the synthesized palladium complexes with AR, aiming to elucidate their potential as modulators of androgen receptor activity and their contribution to the observed cytotoxic effects.

## 2 Results and discussion

### 2.1 Synthesis of Pd complexes 2a–2c

**SCHEME 1:**
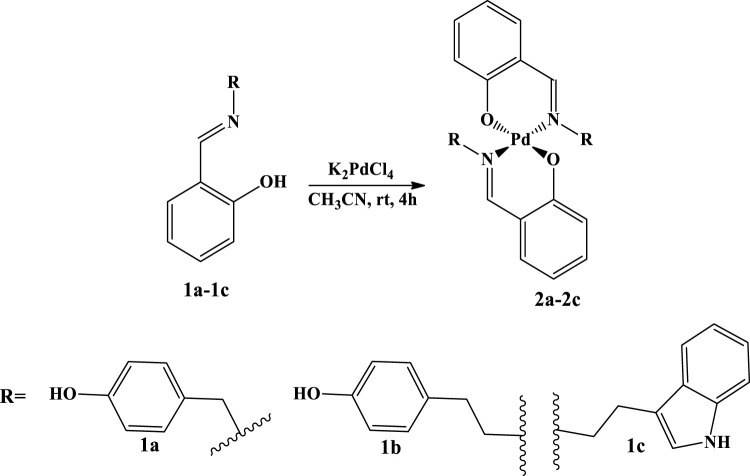
Synthesis of Schiff base palladium (II) complexes **2a–2c**.

The targeted Pd(II) complexes **2a–2c** have been prepared in a one-pot procedure comprising the reaction between corresponding imine **1a–1c** as ligands and K_2_PdCl_4_ as a metal source in acetonitrile, at room temperature in a 1:1 ratio (ligand vs. metal salt), as indicated in Scheme 1. The ligands **1a–1c** were synthesized and structurally characterized in our previous studies (Babić et al., 2025; Marjanović et al., 2024). Besides the salicylic part, the ligands were composed of imine patterns–tryptamine, tyramine, and *p*-OH benzyl moiety, structurally related to the tyramine molecular scaffold. All complexes are new compounds obtained in the form of powders and fully characterized by the IR, ^1^H NMR, ^13^C NMR, mass spectrometry, and elemental analysis (Experimental part, Supplementary Material).

### 2.2 NMR studies

**FIGURE 1:**
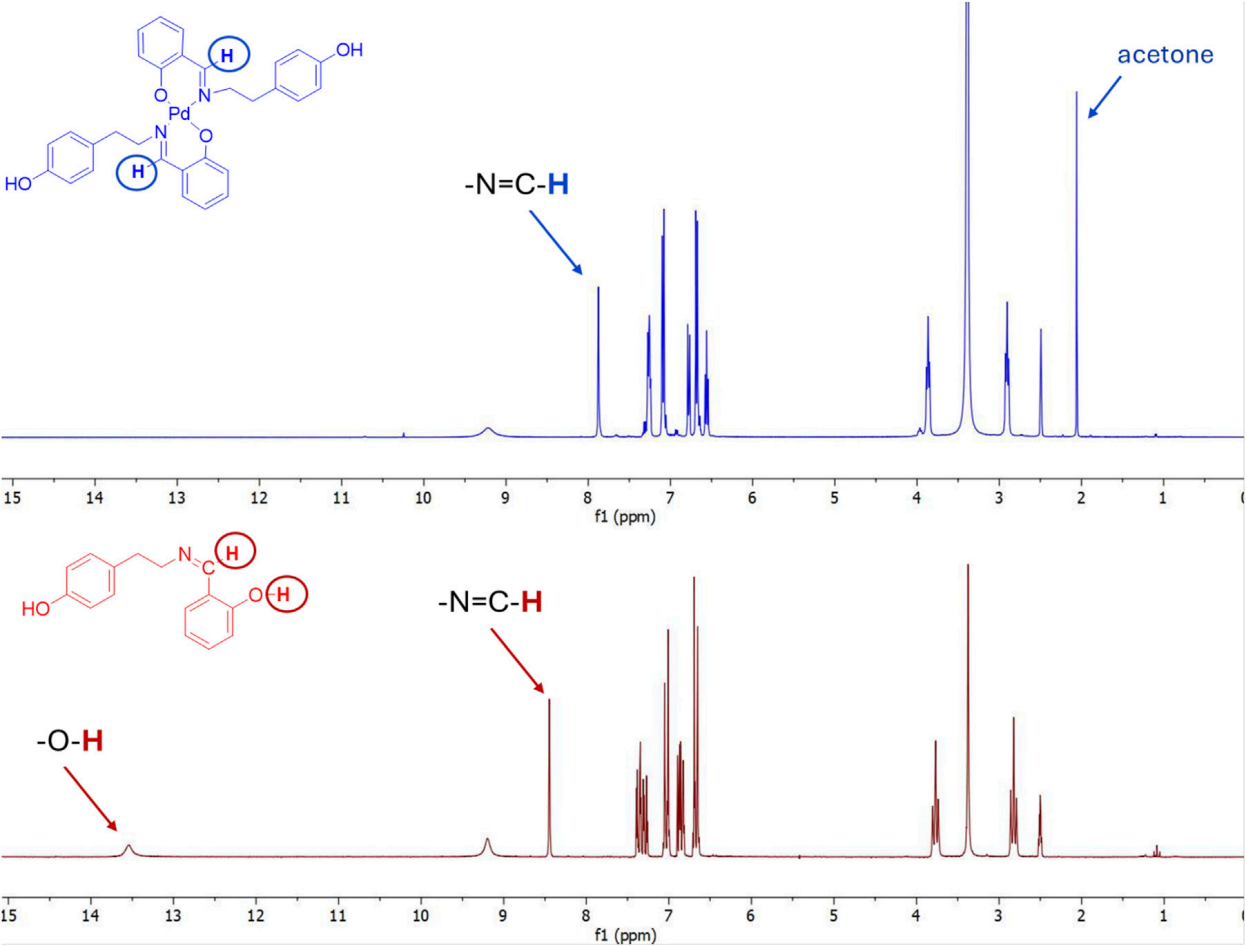
Comparison of ^1^H NMR spectra of starting ligand **1b** and complex compound **2b**.

A detailed overview of the NMR characterization data for all synthesized compounds is provided in the Materials and Methods section, as well as in the Supplementary Material (SI), while representative examples are discussed herein to highlight key structural changes upon complexation. The representative ^1^H NMR spectra of palladium complex **2b** were analyzed by comparison with the spectra of ligand compound **1b**, as depicted in Figure 1. The azomethine proton–**CH = N** from the Schiff base located at 8.45 ppm is, in the case of the palladium complex, shifted to 7.88 ppm, confirming the involvement of the imino group in the coordination with the palladium metal ion. The–OH proton peaks from the salicylic moiety appearing at 13.54 ppm in the ligand spectra completely disappear in the case of palladium complex spectra, undoubtedly confirming the coordination of the palladium ion with the phenolate anion. Additionally, subtle changes in the aromatic region (6.5–8.0 ppm) further support coordination-induced electronic redistribution. The multiplet pattern of aromatic protons becomes slightly broadened and shifts marginally, which can be attributed to changes in magnetic environment and restricted rotation upon complexation. Taken together, these spectroscopic features strongly support the formation of a stable square planar Pd(II) complex, in which the ligand coordinates through both imine nitrogen and deprotonated phenolic oxygen, in agreement with the proposed structure shown in Figure 1.

**FIGURE 2:**
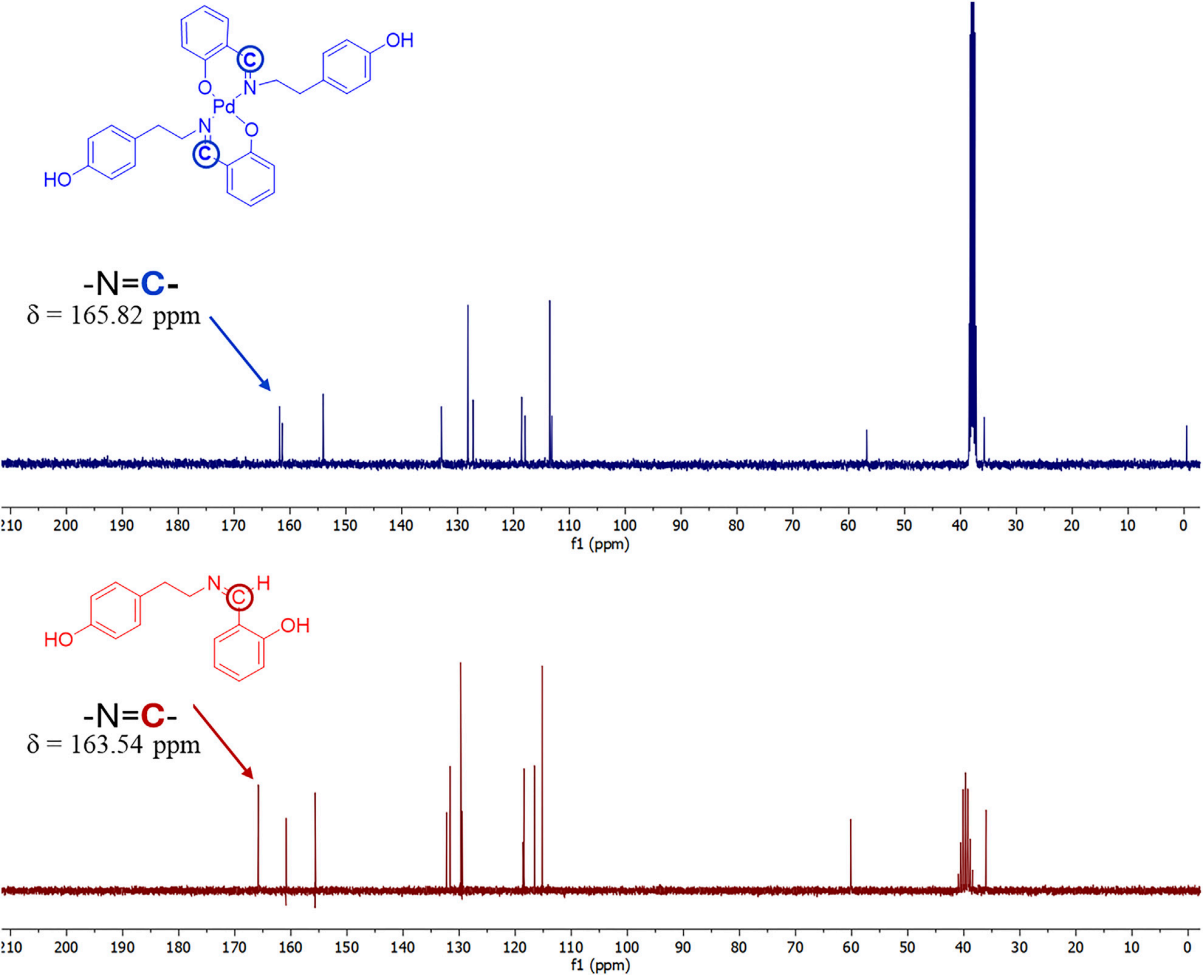
Comparison of ^13^C NMR spectra of starting ligand **1b** and complex compound **2b**.

In the ^13^C NMR spectra, clear changes in the chemical shifts of characteristic carbon atoms were observed upon complexation with Pd(II), supporting the proposed coordination mode. In the free ligand 1b, the signal for the azomethine carbon (–CH = N) appears at 165.82 ppm, while the phenolic carbon bearing the–OH group (–C–OH) resonates at 155.68 ppm (Figure 2). Upon complex formation, the azomethine carbon signal shifts slightly upfield to 163.54 ppm, indicating a decrease in electron density due to the coordination of the imine nitrogen to the palladium center, which is consistent with the formation of a Pd–N bond. The phenolic carbon signal shows a negligible shift (from 155.68 ppm to 155.72 ppm), which suggests that while deprotonation of the hydroxyl group occurs, the local electronic environment of the carbon remains largely stabilized, possibly due to resonance delocalization within the chelate ring and Pd–O bond formation. Additionally, several aromatic carbon signals appear in the 120–140 ppm region, with slight shifts compared to the free ligand, reflecting electronic redistribution upon coordination. These observations provide further evidence of ligand binding and the resulting structural reorganization. Overall, the ^13^C NMR data, in conjunction with the ^1^H NMR results, support the successful coordination of ligand 1b to Pd(II) through the imine nitrogen and phenolate oxygen atoms in complex **2b**. The corresponding ^1^H NMR spectral data for the CH = N and–C-OH protons, as most relevant shifts in complex structure determination for all complexes **2a–2c,** are given in Table 1.

**TABLE 1:** ^1^H NMR spectral data of ligands **1a–1c** and complex compounds **2a–2c**.

Compound	Imine proton-HC = N	Salicylic OH proton
1a	8.65 ppm, s	13.56 ppm, s
2a	8.20 ppm, s	missing
1b	8.45 ppm, s	13.54 ppm, s
2b	7.88 ppm, s	missing
1c	8.48 ppm, s	13.69 ppm, s
2c	7.96 ppm, s	missing

### 2.3 IR analysis

The experimental IR spectra of all investigated ligands and their corresponding Pd(II) complexes are provided in the Supplementary Material (SI). IR spectral analysis offers valuable insights into the coordination behavior of ligands during complex formation with Pd(II) ions. One of the most significant vibrational bands in the FTIR spectra of Schiff base ligands is the imine stretching band (νC = N), typically observed in the range of 1,610–1,650 cm^-1.^ This functional group plays a key role in metal coordination, and any shift in its position upon complexation serves as strong evidence of such interaction. The extent of the νC = N shift correlates with the strength of azomethine nitrogen coordination to the Pd(II) center, further supporting its direct involvement in complex formation. The uniform trend observed across all examined systems confirms that the ligands coordinate with the palladium ion via the imine nitrogen. The FTIR spectral data for free ligands (**1a–1c**) and Pd(II)-complexes **(2a–2c)** are given in Table 2, and these spectral features collectively confirm the bidentate coordination mode of the Schiff base ligand via the imine nitrogen and phenolic oxygen atoms to the Pd(II) center. Upon coordination of palladium via the nitrogen’s imino group, the strong C=N stretching band shifts to lower wavenumbers, indicating a weakening of the bond due to donation of the nitrogen lone pair to Pd(II). Also, the appearance of two new weak bands, attributed to Pd–N stretching and Pd–O stretching vibrations, affirms the formation of complexes. These IR findings are consistent with the ^1^H and ^13^C NMR data previously discussed, providing complementary experimental evidence for the formation of bidentate N, O-chelated palladium (II) complexes.

**TABLE 2:** FTIR spectral data for ligands **1a–1c** and Pd(II)-complexes **2a–2c**.

Compound	ν_(C=N)_/cm-1	ν_(Pd-N)_/cm-1	ν_(Pd-O)_/cm-1
1a	1,641	−	−
1b	1,631	−	−
1c	1,631	−	−
2a	1,615	513	461
2b	1,611	506	460
2c	1,618	559	453

### 2.4 Mass spectrometry

The electrospray ionization mass spectrometry (ESI-MS) analysis was performed to confirm the molecular composition and stoichiometry of the synthesized palladium (II) complexes. In the mass spectrum of complex **2a** (Mol.Wt 558.08), a dominant signal was observed at *m/z* = 559.0857, which corresponds to the protonated molecular ion [M + H] ^+^, confirming the formation of the proposed bis-Schiff base Pd(II) complex (ESI, Supplementary Figure SI13). Similarly, for complex **2c** (Mol.Wt 632.14), a strong peak at *m/z* = 633.1490 was detected, also corresponding to the [M + H]+species (ESI, Supplementary Figure SI22), while the spectra of compound **2b** (Mol.Wt 586.11) revealed the peak at 587.03 (ESI, Supplementary Figure SI18). The presence of these molecular ion peaks, which match the calculated molecular masses of the respective complexes, confirms the proposed coordination of two Schiff base ligands to the Pd(II) ion in a 2:1 ligand-to-metal ratio. These results provide direct evidence for the successful formation of the target Pd(II) complexes and are in full agreement with the spectroscopic data (NMR and IR), thereby supporting the proposed coordination environment illustrated in the structural models.

### 2.5 Molar conductivity

The molar conductivity of complexes **2a–2c** was measured in DMSO and demonstrated values ranging from 15.4 16.3–17.2 S cm2 mol^-1^, respectively. This is in agreement with the reports where the palladium is coordinated with two ligand compounds forming complexes that are neutral in nature and that behave as nonelectrolytes in solution (Ali et al., 2013; Geary, 1971).

### 2.6 Absorption spectroscopy

**FIGURE 3:**
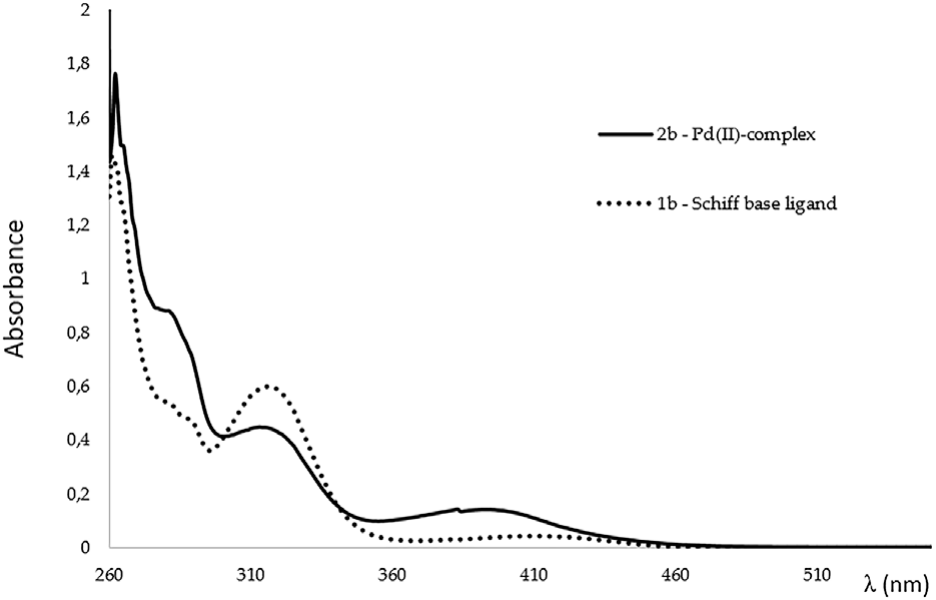
UV-Vis spectra of the Schiff base **1b** (‧‧‧) and its Pd(II)-complex **2b** (−).

UV-Vis spectra were recorded in DMSO as a solvent, at room temperature, and the data of the absorption wavelength maxima (λ max) are given in Table 3 and Figure 3. Both Schiff base **1b** and its Pd(II) complex **2b** exhibited strong bands corresponding to π → π* aromatic transitions. Further, two more bands are attributed to the ligand **1b**, the medium intensity π → π* and very weak and broad n→π* transition of the imine group. Upon coordination, the π → π* band of the imine group decreases in intensity, while the new band at 393 nm, attributed to Ligand-to-Metal Charge Transfer (LMCT), appears (Felicio et al., 2001; Hussain et al., 2019). The stability of Schiff base metal complexes is a key factor influencing their performance during biological evaluations. Unstable complexes, prone to hydrolysis or degradation, may lead to reduced efficacy, altered mechanisms of action, or false-positive/negative outcomes. Palladium (II), typically adopting a square-planar geometry (Kostić et al., 2003), forms stable complexes with bidentate Schiff base ligands, but its coordination environment can be sensitive to ligand exchange. Considering that Pd(II) is a soft Lewis acid and has affinity for soft donor atoms (like sulfur) it was necessary to evaluate the stability of synthetized complexes in DMSO, as all stock solutions for biological experiments were prepared in DMSO as a solvent. Possible ligand exchange may lead to complex decomposition or formation of Pd–DMSO adducts. In order to determine the stability of selected complex **2b** in DMSO, a series of UV-Vis spectra in the range of 200–600 nm were recorded after t = 0, 24, and 48 h (Supplementary Figure SI17) (Marjanović et al., 2023). Spectroscopic data confirms the preservation of the complex’s stability in DMSO, retaining its structural integrity without signs of ligand displacement or decomposition.

**TABLE 3:** UV-visible spectral data for the Schiff base **1b** and its Pd(II)-complex **2b**.

Compound	Λ (nm)	Band asignment
1b	262, 282, 290	π→π* aromatic
	317	π→π* imine
	410	π→π* imine
2b	262, 282, 290	π→π* aromatic
	313	π→π* imine
	393	LMCT

### 2.7 Stability assay

As one of the essential features of compounds with drug-like potential is their stability in a physiological environment, the stability screening should be an integral part of the comprehensive assessment of a compound’s pharmaceutical properties. For this assay, compound **2b** was selected as a representative. In Supplementary Figure SI23, time-dependent UV-Vis absorption spectra of complex **2b** are presented. Tested compounds have shown sufficient stability in solution under physiologically relevant conditions, where slight changes may be attributed to the solvent evaporation as absorbance slightly decreases over time.

### 2.8 Viscosity measurement

To examine the nature of the interaction of the obtained compound **2c** and deoxyribonucleic acid sodium salt from calf thymus (CT-DNA), the viscosity measurements were done. The viscosity of DNA is sensitive to length changes, and it is regarded as the least ambiguous and the most critical clue of DNA binding mode in solution (Li et al., 2010). As the intercalation strength is usually proportional to the increase in the viscosity of the DNA, from the data presented in Supplementary Figure SI24, it can be concluded that compound **2c** intercalates very weakly with the DNA strands, as the addition of increasing concentrations of **2c** to the DNA solution led to a moderate increase in viscosity. These data indicate that the interaction of complex **2c** with DNA is with a strong preference for the minor groove binding rather than intercalation, which is in accordance with the competitive fluorescence quenching studies.

### 2.9 *In vitro* cytotoxic study

**FIGURE 4:**
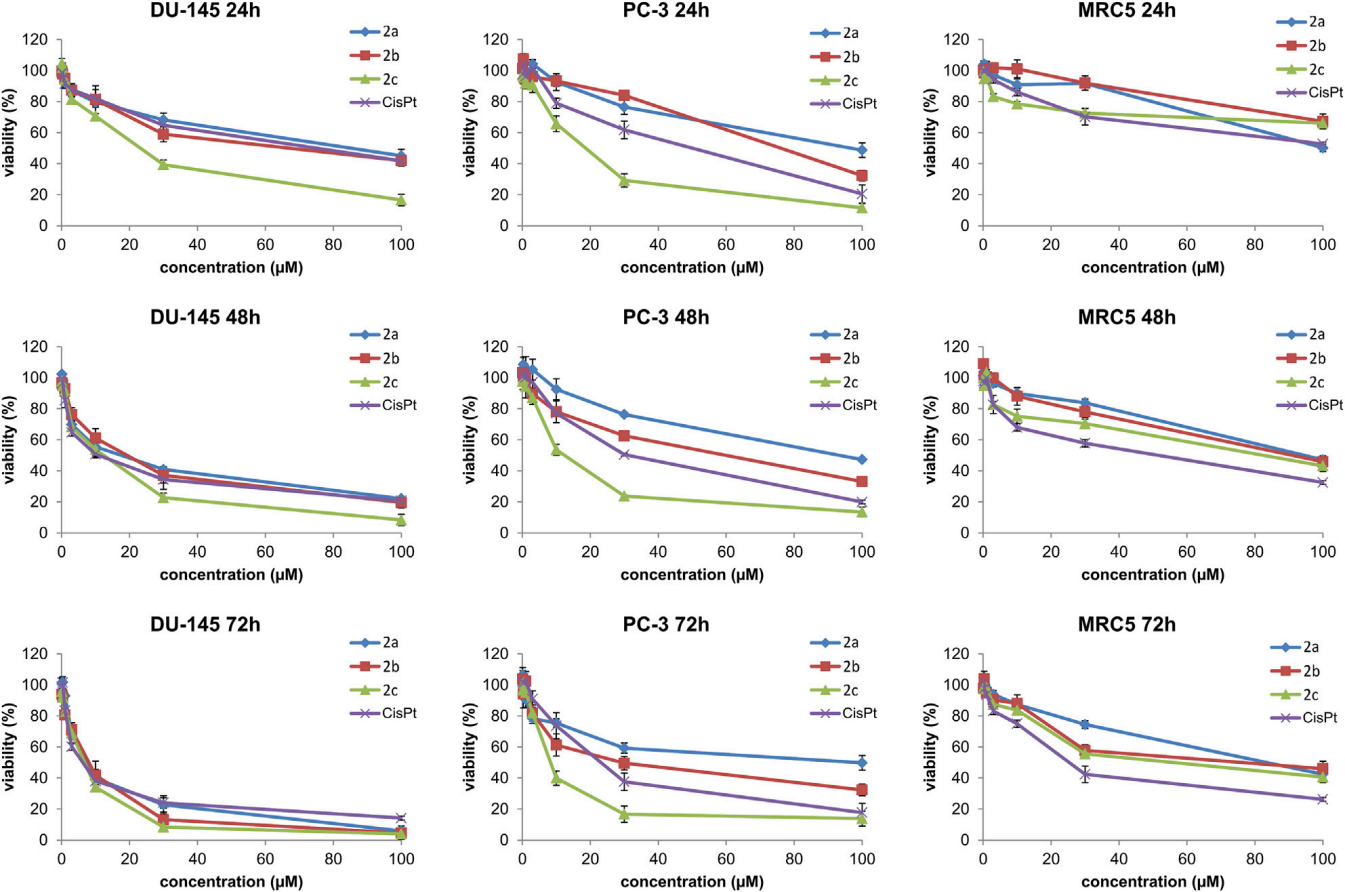
The effects of Pd(II) complexes (**2a–2c**) and cisplatin (CisPt) on the viability of human prostate carcinoma cells without prostate-specific membrane antigen (PSMA) and androgen receptor-independent (DU-145), human prostate carcinoma cells (PC-3), and human non-tumor cells (MRC-5).

The cytotoxicity of the three synthesized Pd(II) complexes (**2a–2c**) was assessed *in vitro* using human prostate carcinoma cells lacking prostate-specific membrane antigen (PSMA) and androgen receptor independence (DU-145), human prostate carcinoma cells (PC-3), and non-cancerous human fibroblasts (MRC-5). It is important to note that PSMA receptors are extensively used in prostate cancer research (Klika et al., 2024). The evaluation was conducted via the MTT assay following 24, 48, and 72 h of treatment. For comparative purposes, the cytotoxic profile of cisplatin was also examined. The findings unequivocally demonstrate that all Pd(II) complexes, as well as cisplatin, exhibit dose-dependent cytotoxic effects against DU-145 and PC-3 cell lines. The IC_50_ values for these Pd(II) complexes and cisplatin are summarized in Table 4, reinforcing their potential as anticancer agents (Figure 4).

**TABLE 4:** IC_50_ values in µM for Pd(II) complexes **2a–2c** and cisplatin (CP) after 24, 48, and 72 h of drug exposure. Results are presented as mean ± SD and determined from the results of MTT assay in three independent experiments.

Cell line	Time	2a	2b	2c	CP
DU-145	24 h	83.6 ± 9.2	75.9 ± 7.3	48.4 ± 5.1	22.3 ± 2.4
	48 h	17.5 ± 1.6	19.2 ± 2.1	11.0 ± 1.2	11.4 ± 1.1
	72 h	9.3 ± 0.9	8.7 ± 0.8	7.1 ± 0.8	8.2 ± 0.8
PC-3	24 h	94.1 ± 9.1	75.2 ± 7.2	38.4 ± 3.6	15.6 ± 1.4
	48 h	89.8 ± 8.8	58.6 ± 5.6	12.5 ± 1.1	29.7 ± 3.1
	72 h	79.3 ± 7.7	29.1 ± 2.8	8.6 ± 0.9	21.9 ± 2.2
MRC-5	24 h	94.3 ± 9.7	87.7 ± 8.8	80.7 ± 8.1	90.1 ± 9.2
	48 h	92.4 ± 9.1	78.5 ± 7.7	75.6 ± 7.7	41.9 ± 4.4
	72 h	83.0 ± 8.1	49.2 ± 5.1	42.3 ± 4.1	24.4 ± 0.8

**FIGURE 5:**
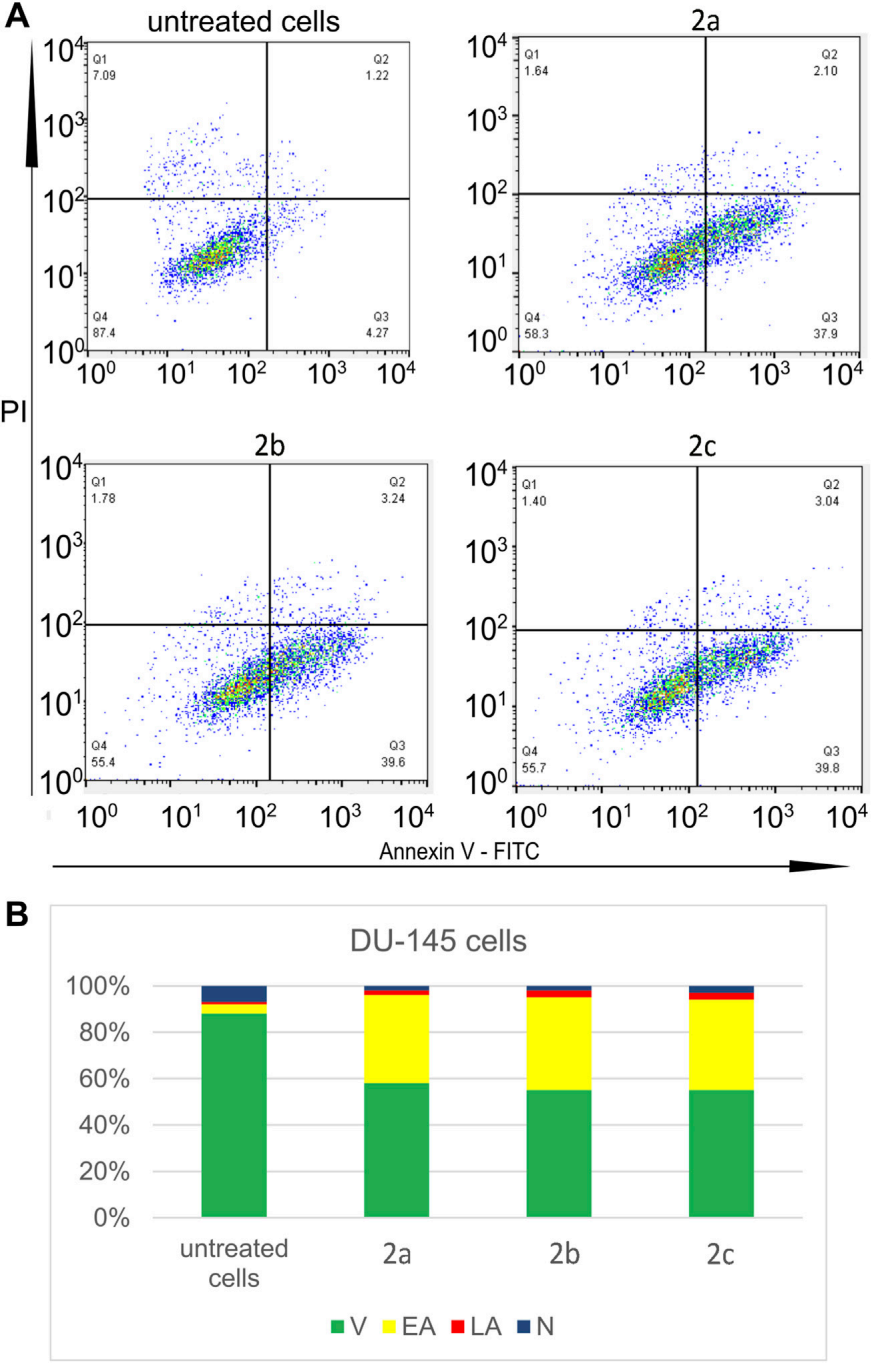
Three tested Pd(II) complexes (**2a–2c**) decrease viability of treated human prostate carcinoma cells without prostate-specific membrane antigen (PSMA) and androgen receptor-independent (DU-145); **(A)** Representative flow-cytometry plots using Annexin V-FITC/PI staining for apoptosis. **(B)** The average percentage of DU-145 viable cells (V), early apoptotic cells (EA), late apoptotic cells (LA) and necrotic cells (N) after 24 h treatment with IC_50_ of Pd(II) complexes (**2a–2c**).

**FIGURE 6:**
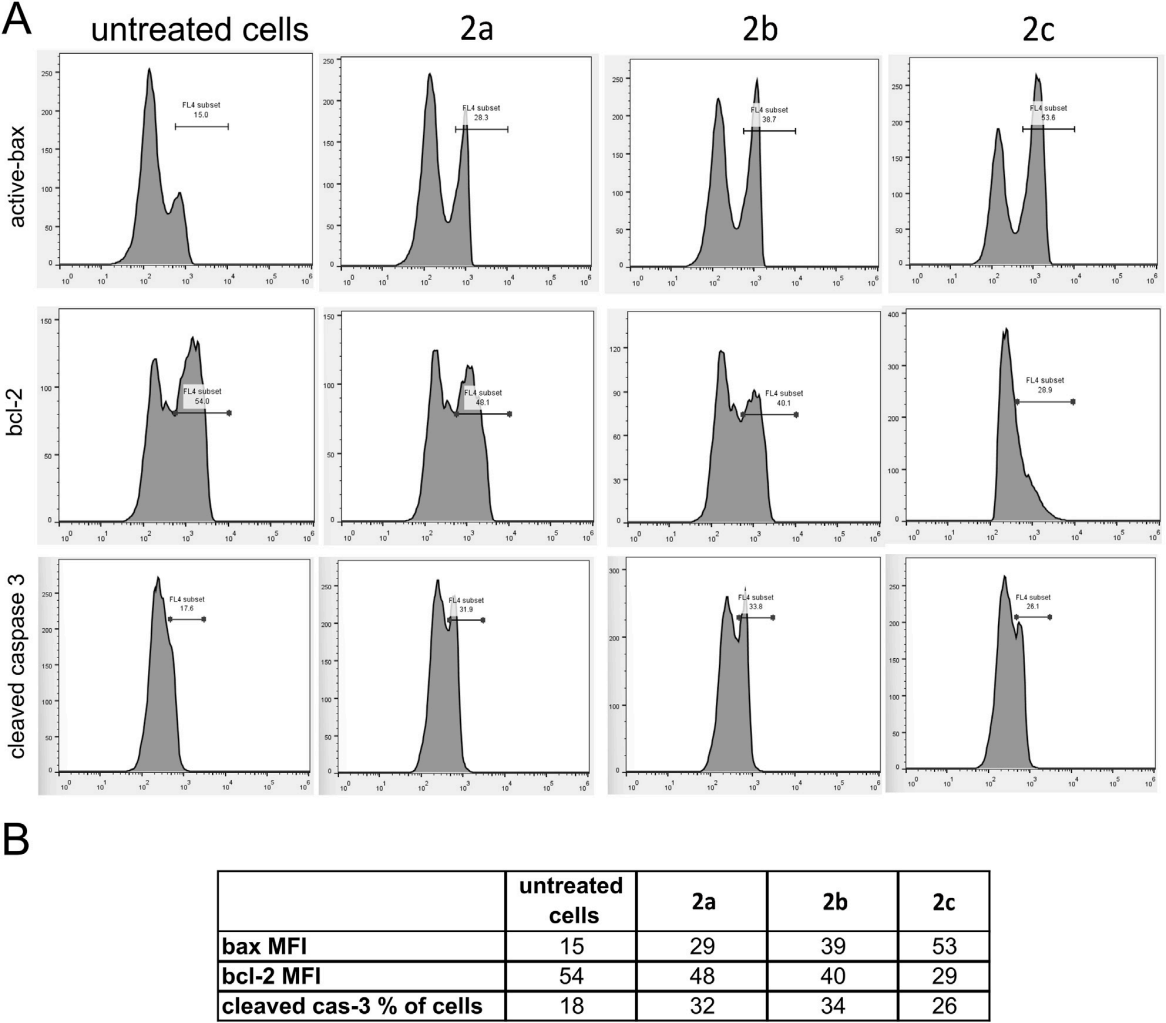
Pd(II) complexes **2a–2c** induce apoptosis of human prostate carcinoma cells without prostate-specific membrane antigen (PSMA) and androgen receptor-independent (DU-145) via a caspase-dependent pathway. **(A)** MFI values (mean fluorescence intensity) for anti-apoptotic protein bcl-2, proapoptotic protein Bax and cleaved caspase 3 of DU-145 cells treated with IC_50_ of Pd(II) complexes **2a–2c**. **(B)** Overview of MFI values for pro-apoptotic protein active bax, antiapoptotic bcl-2 and active (cleaved) caspase-3 of DU-145 cells treated with IC_50_ of Pd(II) complexes **2a–2c**.

All tested Pd(II) complexes, as well as cisplatin, significantly reduced the viability of DU-145 and PC-3 prostate carcinoma cell lines following 24, 48, and 72 h of treatment. Among the synthesized complexes, compound **2c** exhibited the most pronounced cytotoxic activity across all exposure durations and against both cell lines. Notably, the cytotoxic effect of complex **2c** surpassed that of cisplatin after 48 and 72 h of treatment under identical experimental conditions (Figure 4; Table 4). Furthermore, while all Pd(II) complexes demonstrated a relatively lower cytotoxic impact on non-cancerous human fibroblasts (MRC-5) compared to cisplatin, exceptions were observed for complexes **2b** and **2c** at the 24-h mark. These findings underscore the selective anticancer potential of the newly synthesized Pd(II) complexes (**2a–2c**), particularly their preferential activity against human prostate cancer cell lines. Although compounds **2b** and **2c** exhibited moderate cytotoxicity toward MRC-5 fibroblasts at 24 h, their activity against prostate cancer cell lines significantly increased at 48 h and 72 h, resulting in improved selectivity over time. For example, at 72 h, compound **2c** showed IC_50_ values of 7.1 µM (DU-145) and 8.6 µM (PC-3) compared to 42.3 µM in MRC-5, suggesting a preferential cytotoxic effect on malignant cells. In contrast, cisplatin demonstrated potent cytotoxicity across all tested cell lines, with only limited differences between cancerous and non-cancerous cells (e.g., 8.2 µM in DU-145 vs. 24.4 µM in MRC-5 at 72 h), indicating lower selectivity. These findings highlight the potential therapeutic advantage of compounds **2b** and **2c**, particularly **2c**, which combines increased anticancer activity with reduced impact on non-tumorigenic cells over extended exposure times. The subsequent phase of our investigation focused on determining the mode of cell death induced by the Pd(II) complexes in DU-145 and PC-3 prostate carcinoma cells. To achieve this, an annexin V/PI staining assay was employed. The results revealed that all tested Pd(II) complexes effectively triggered apoptosis (Figure 5). Notably, in DU-145 cells, treatment with an IC_50_ dose of complex **2c** resulted in the highest proportion of necrotic cells. Further analyses were conducted to investigate whether these Pd(II) complexes affected the intracellular levels of the anti-apoptotic protein Bcl-2 and the pro-apoptotic protein Bax. Additionally, caspase-3 activation was assessed in DU-145 cells exposed to the Pd(II) complexes. The findings demonstrated a significant reduction in Bcl-2 levels and a concomitant increase in BAX concentrations in cells treated with IC_50_ doses of the Pd(II) complexes compared to untreated controls (Figure 5). Moreover, a marked elevation in the percentage of cells exhibiting active caspase-3 was observed following treatment with the Pd(II) complexes (Figure 6). Collectively, these results indicate that the Pd(II) complexes (**2a–2c**) decreased the Bcl-2/BAX ratio, thereby promoting caspase-3 activation and inducing apoptosis via intrinsic apoptotic pathways.

**FIGURE 7:**
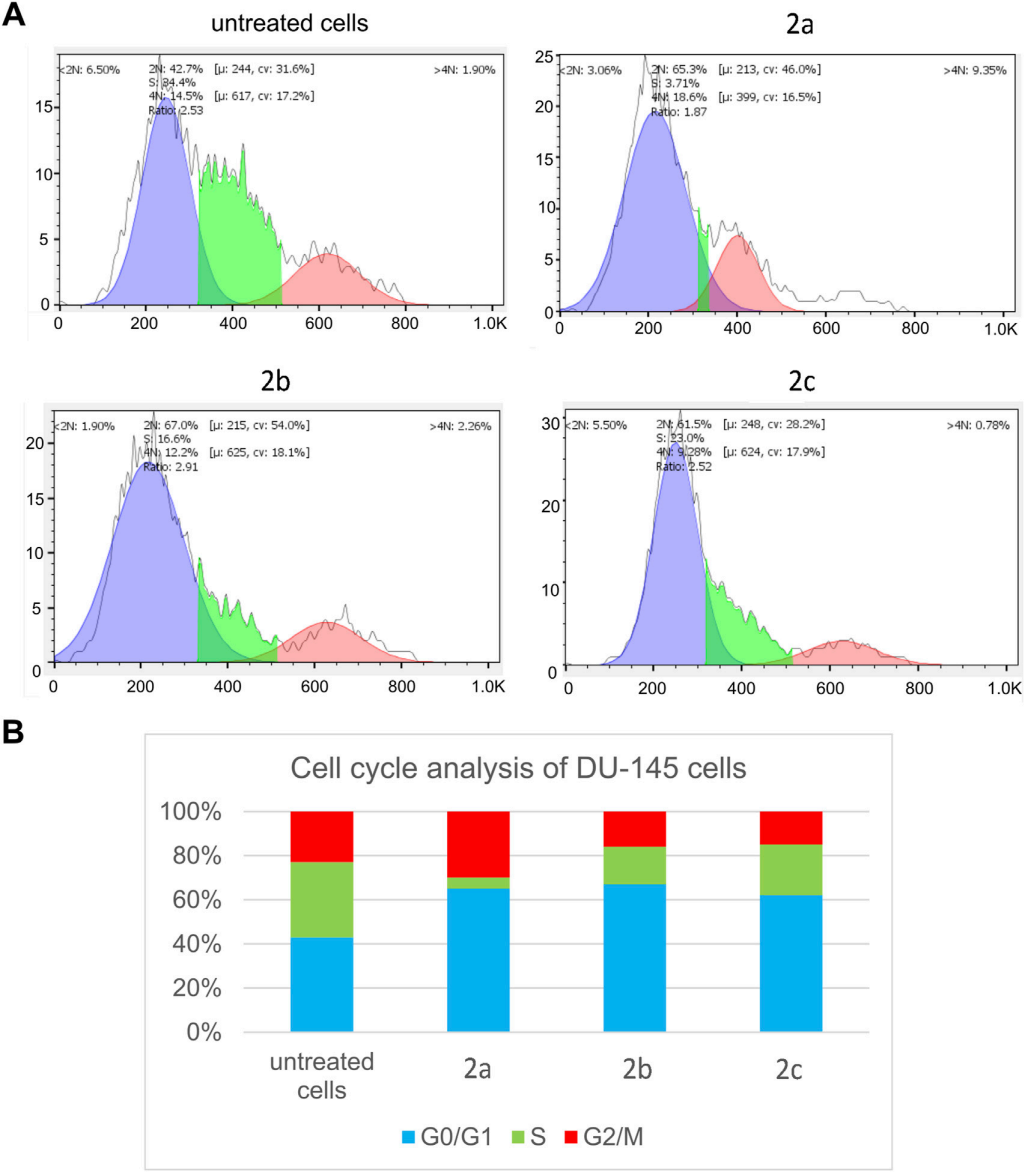
Pd(II) complexes (**2a–2c**) induce G2/M cell cycle arrest of treated tumour DU-145 cells. **(A)** Representative flow-cytometry plots using PI staining for cell cycle phase detection. **(B)** The average percentage of DU-145 cells in G0/G1 cell cycle phase (blue), S cell cycle phase (green) and G2/M cell cycle phase (red) after 24 h treatment with IC_50_ of Pd(II) complexes (**2a–2c**).

The induction of apoptosis and/or cell cycle arrest is a well-established mechanism for reducing cancer cell viability (Kacar et al., 2017; Deljanin et al., 2016). Notably, cancer cells exhibit a heightened reliance on DNA damage repair mechanisms and the abrogation of the G2 checkpoint. Arrest in the G0/G1 phase typically halts cellular proliferation while allowing for the repair of damage caused by anticancer agents. In contrast, G2/M phase arrest is often associated with the initiation of apoptosis. In our study, we investigated the effects of IC_50_ concentrations of Pd(II) complexes **2a–2c** on the cell cycle of DU-145 prostate cancer cells 24 h post-treatment. Flow cytometric analysis was performed on propidium iodide (PI)-stained cells to evaluate cell cycle distribution (Figure 7). The results revealed that all Pd(II) complexes induced G0/G1 phase arrest in DU-145 cells, highlighting their ability to interfere with cell cycle progression. Furthermore, variations in the mechanisms of action among the Pd(II) complexes (**2a–2c**) were observed, underscoring their distinct biological activities.

**FIGURE 8:**
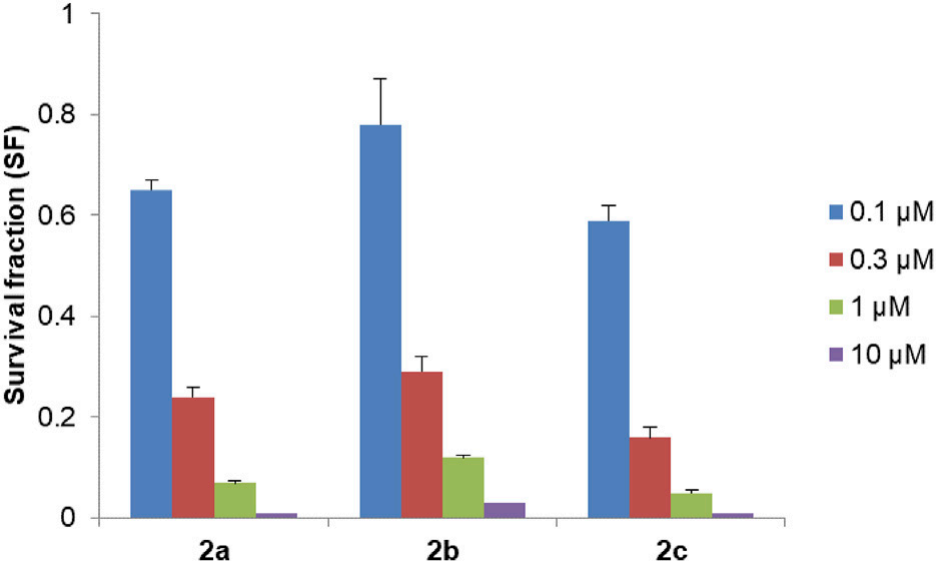
Survival fraction (SF) for Pd(II) complexes **2a–2c** tested in four different concentrations in DU-145 cells. Results are presented as mean ± standard deviation.

The long-term antiproliferative effect of Pd(II) complexes was tested by using a clonogenic assay. All tested compounds showed a significant antiproliferative effect, which was dose-dependent, and the most pronounced effect was detected after treatment with complex **2c** (Figure 8).

**FIGURE 9:**
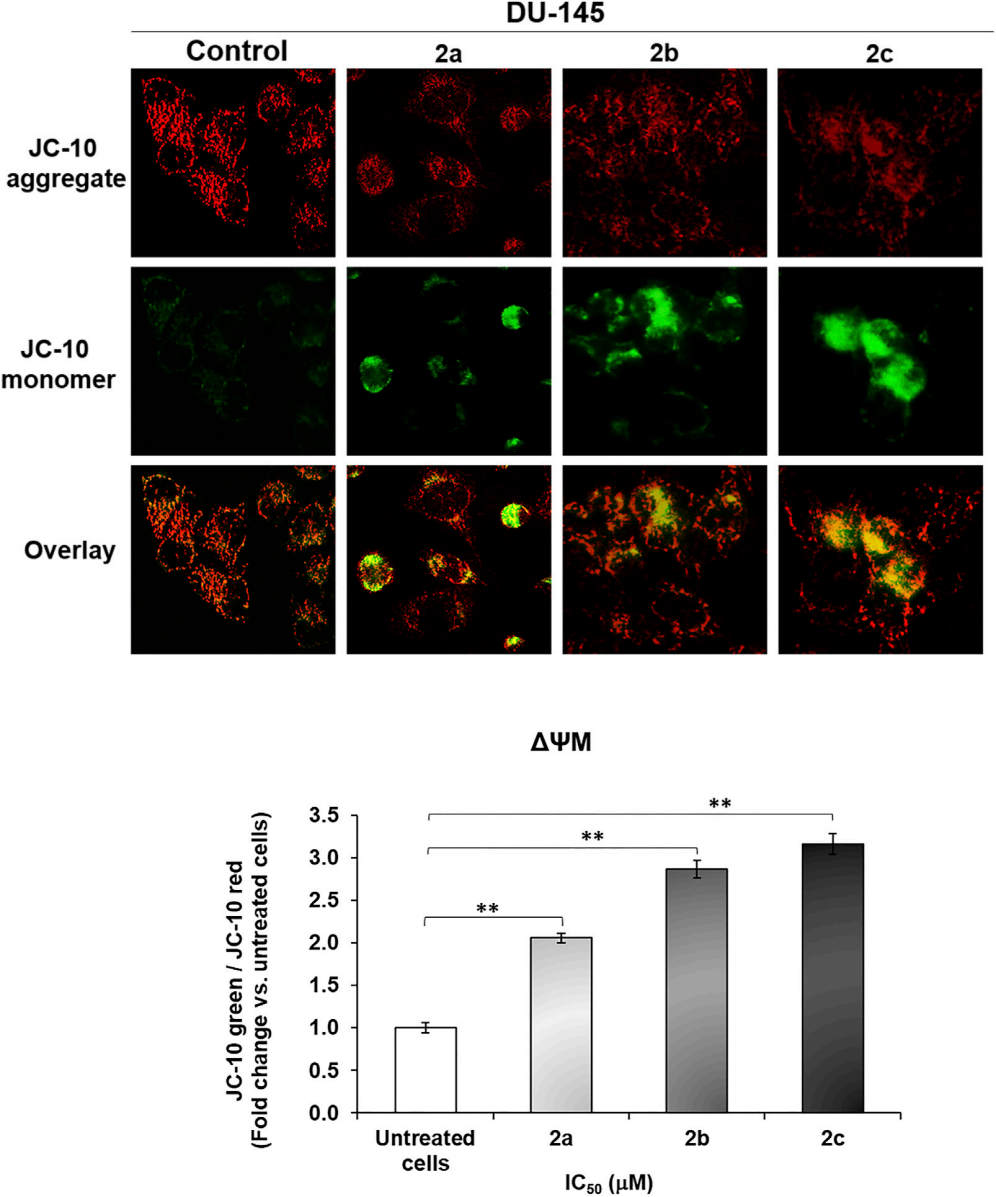
Palladium complexes **2a–2c** induce changes in mitochondrial membrane potential (ΔΨM) in DU-145 cells. DU-145 cells were treated with palladium complexes **2a–2c** (IC_50_-μM) for 48 h and stained with the mitochondrial fluorescent probe JC-10. Pd complexes reduced the red fluorescence of the JC-10 aggregate within the mitochondria while increasing the green fluorescence of the JC-10 monomer in the cytoplasm. The MFI of the red and green forms of JC-10 was measured using ImageJ. The histogram shows the mean fold change of the MFI JC-10 green/MFI JC-10 red ratio. The bars show the mean ± SE; (N = 3). Significance levels are indicated as **p < 0.01 compared to control. Scale bar: 100 μm; magnification: ×40.

Different palladium complexes have demonstrated the ability to induce apoptosis by activating the intrinsic mitochondrial pathway (Czarnomysy et al., 2021). Mitochondria combine death signals from both intrinsic and extrinsic apoptotic pathways. The dysregulation of mitochondrial membrane potential (ΔΨM) is crucial in the activation of mitochondrial apoptosis. We examined the capacity of palladium complexes **2a–2c** to induce mitochondrial apoptosis in DU-145 cells by assessing changes in ΔΨM using the fluorescent dye JC-10. In living cells, JC-10 mainly produces red fluorescent aggregates within the mitochondrial matrix. In contrast, in dying cells, it mainly diffuses from the mitochondria into the cytoplasm, leading to the production of a green monomeric form. Treatment with complexes **2a–2c** resulted in a significant rise in JC-10 green fluorescence and a reduction in JC-10 red fluorescence, indicating a loss of ΔΨM. Specifically, our results demonstrated that all tested compounds significantly increased the JC10 green/JC-10 red ratio by 2.1-, 2.9-, and 3.2-fold, respectively, compared to untreated cells (Figure 9). These findings suggest that the complexes **2a–2c** effectively induce apoptosis in DU-145 cells via the intrinsic mitochondrial pathway by disrupting ΔΨM.

The query outlines the synthesis and evaluation of three new Pd(II) complexes (**2a–2c**) for their cytotoxicity and mechanisms of action against human prostate carcinoma cells (DU-145 and PC-3) and non-cancerous fibroblasts (MRC-5). The findings demonstrate that all three complexes exhibit potent and selective cytotoxic effects on prostate carcinoma cells, with complex **2c** showing superior efficacy against DU-145 cells after 72 h compared to cisplatin. The cytotoxic potency follows the order **2a < 2b < 2c**, potentially due to the presence of an indole ring in the ligand of complex **2c** (Kandeel et al., 2025; Kamel et al., 2019). Conversely, the selectivity increases in the reverse order (**2c** < **2b** < **2a**), with all Pd(II) complexes displaying greater selectivity than cisplatin.

The search results corroborate the potential of Pd(II) complexes as anticancer agents. For instance, studies have shown that Pd(II) complexes induce apoptosis through mechanisms such as mitochondrial membrane depolarization, Bax upregulation, and Bcl-2 downregulation in prostate cancer cells like DU-145 and PC-3 (Valentini et al., 2009; Ulukaya et al., 2013). Additionally, some Pd(II) complexes demonstrate selective cytotoxicity against cancer cells while sparing non-cancerous cells. The observed differences in efficacy and selectivity among Pd(II) complexes may be attributed to structural variations in their ligands, as highlighted in other research on similar compounds. These findings underscore the therapeutic potential of Pd(II) complexes for prostate cancer treatment.

Pd(II) complexes exhibit substantial anticancer potential against DU-145 human prostate cancer cells, primarily through the induction of apoptosis, particularly early apoptosis (Plutín et al., 2014). This mechanism was confirmed in our study, where treatment with Pd(II) complexes resulted in a significant transition of cells from viability to the early apoptotic stage. These complexes demonstrate both cytotoxic and antiproliferative properties by disrupting cellular metabolism and inducing cell death (Valentini et al., 2009). Furthermore, Pd(II) complexes exert both short-term and long-term antiproliferative effects, significantly impairing cell growth and abolishing long-term proliferation. Their action also includes inhibition of cell migration and adhesion, induction of morphological changes such as cellular shrinkage, and interaction with DNA, leading to double-stranded DNA cleavage–a key contributor to their cytotoxic effects (Laginha et al., 2023).

Studies corroborate these findings, highlighting that Pd(II) complexes induce apoptosis via mitochondrial membrane depolarization, Bax upregulation, and Bcl-2 downregulation (Carneiro et al., 2020). Additionally, their ability to disrupt DNA integrity without activating DNA repair enzymes further emphasizes their therapeutic potential (Sharma et al., 2016). These results collectively position Pd(II) complexes as promising candidates for prostate cancer treatment due to their multifaceted anticancer mechanisms.

Research has demonstrated that Pd(II) complexes can induce apoptosis through multiple pathways, including the activation of cell death receptors and the generation of reactive oxygen species (ROS). These complexes have shown significant efficacy against DU-145 prostate cancer cells, highlighting their potential as anticancer agents. The metabolic effects triggered by Pd(II) complexes can be more rapid compared to those of cisplatin, which may result in faster patient recovery. This rapid metabolic response is advantageous as it could potentially reduce the duration and severity of side effects associated with chemotherapy.

Studies on various Pd(II) complexes have revealed diverse mechanisms of action, including DNA interaction, ROS-mediated mitochondrial dysfunction, and the activation of intrinsic and extrinsic apoptotic pathways (Carneiro et al., 2020; Espino et al., 2020). The ability of Pd(II) complexes to induce apoptosis without relying on DNA damage, as seen with cisplatin, suggests they may offer a distinct therapeutic approach with potentially fewer side effects (Espino et al., 2020). Additionally, the activation of cell death receptors and the induction of apoptosis through ROS production underscore the versatility of Pd(II) complexes in targeting cancer cells (Carneiro et al., 2020; Aydinlik et al., 2021).

Palladium (II) complexes have demonstrated significant cytotoxic and growth-inhibitory effects on the DU-145 human prostate adenocarcinoma cell line, primarily through mechanisms such as DNA interaction and apoptosis induction. These complexes induce cellular changes, including reduced cell numbers and morphological alterations, which are indicative of their cytotoxic effects. The formation of DNA adducts following the interaction of Pd(II) complexes with DNA is a key contributor to their anticancer activity. Studies highlight that complexation with thiosemicarbazone ligands enhances the antitumor potential of Pd(II) complexes, making this a promising strategy for developing new anticancer agents. For instance, Pd(II)-thiosemicarbazone complexes have shown potent cytotoxicity against DU-145 cells, with IC_50_ values significantly lower than those of cisplatin (Hernández et al., 2013). Furthermore, Pd(II) complexes have been shown to modulate protein expression in DU-145 cells, potentially influencing pathways critical to cancer progression (Kontek et al., 2011). These findings underscore the therapeutic potential of Pd(II) complexes and the need for further research to optimize their efficacy and selectivity.

### 2.10 DNA interaction study

Many metal-based anticancer agents have DNA as a primary potential biological target. Accordingly, it is very important to understand the binding properties of different transition metal ion complexes. Two possible binding modes of the transition metal ion complexes toward DNA are defined as covalent and non-covalent types of interactions. The covalent bindings consider the replacement of the labile ligand of the complex by a nitrogen base of DNA, and noncovalent are intercalation, electrostatic, or groove binding. The most employed method for determination of binding mode between complexes and DNA is fluorescence quenching (Recio Despaigne et al., 2014). For this investigation competitive studies with EB and Hoechst, 33,258 (Hoe) were made using fluorescence quenching experiments.

**FIGURE 10:**
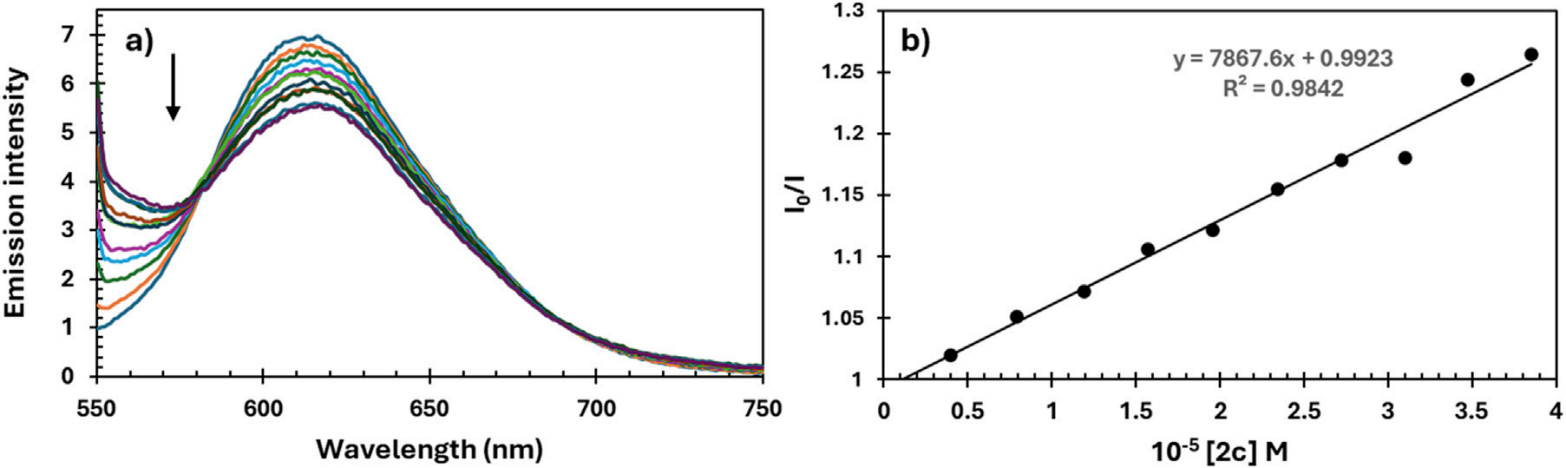
**(a)** Emission spectra of EB-DNA in the absence and presence of complex **2c**. pH = 7.4; λex = 550 nm; **(b)** plots of I_0_/I versus concentration of quencher [**2c**].

Ethidium bromide (EB) is a classical intercalator that gives significant fluorescence emission intensity when it intercalates into the base pairs of DNA. The fluorescence titration of EB-DNA with increasing amounts of **2c** is shown in Figure 10. The decrease in the emission intensity of the band at 611 nm suggests that complex **2c** can replace EB from EB-DNA and interact with DNA by the interactive mode (Tarushi et al., 2010; Tarushi et al., 2013).

The Stern-Volmer quenching constants (K_sv_) are calculated from the slopes of the plots of I_0_/I vs. (**4**) from Equation 1 (Lakowicz and Weber, 1973)

$$I_{0}/I=1+K_{\text{sv}}\left[Q\right]$$ (1)

and presented in Table 5. Quenching rate constants, k_q_ are calculated through the correlation (Klika et al., 2022), since its known that the average fluorescence lifetime of the DNA without a quencher (τ_0_) is 10^–8^ s (Equation 2) (Aydinlik et al., 2021; Lakowicz et al., 1999)

$$K_{\text{sv}}=k_{q}\times \tau_{0}$$ (2)

**TABLE 5:** The bimolecular quenching rate constant (k_q_), Stern–Volmer constant (K_sv_), and correlation coefficient (R) for complex **2c** as a quencher.

Complex	Test	k_q_[M^-1^s^-1^]	K_sv_[M^-1^]	R^2^
2c	EB	0.8 × 1,012	0.8 × 104	0.9842
	Hoe	3.1 × 1,012	3.1 × 104	0.9455

The obtained values for K_sv_ suggest that **2c** can interact with DNA molecules in interactive mode (Tarushi et al., 2013).

**FIGURE 11:**
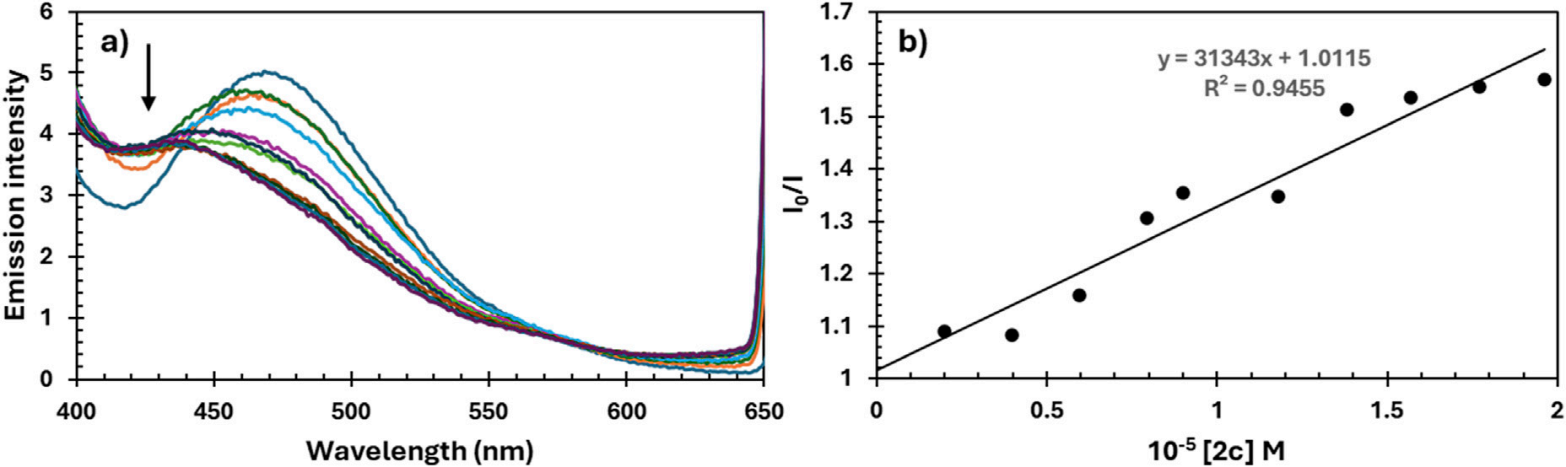
**(a)** Emission spectra of Hoe-DNA in the absence and presence of complex **2c**. pH = 7.4; λex = 346 nm; **(b)** plots of I_0_/I versus concentration of quencher [**2c**].

In order to further elucidate the binding mode of complex **2c** with CT-DNA, competitive fluorescence quenching studies were also conducted using Hoechst 33,258 (Hoe), a synthetic derivative of N-methylpiperazine known to bind specifically within the minor groove of DNA (Vojtek et al., 2019). Minor groove binding represents a non-covalent interaction typical for small-molecule drugs and bioactive compounds, often associated with sequence-selective recognition (Kandeel et al., 2025). The fluorescence titration of the pre-formed Hoe–DNA complex with increasing concentrations of complex **2c** is shown in Figure 11. A gradual decrease in fluorescence intensity suggests that complex **2c** can effectively displace Hoe from the minor groove, indicating a possible groove-binding mode of interaction with DNA.

The decrease in fluorescence intensity, as shown in Figure 11, indicates that the palladium complex **2c** is capable of displacing Hoe from the Hoe–DNA complex. The Stern–Volmer quenching constant (K_sv_), calculated and presented in Table 5, supports this observation and further confirms the proposed interaction between complex **2c** and DNA through minor groove binding.

By comparing the K_sv_ values obtained from the EB and Hoe fluorescence quenching experiments (Table 5), it can be concluded that the complexes interact with double-stranded DNA through both binding modes–intercalation and minor groove binding. However, the higher Stern–Volmer constant observed for the Hoe displacement assay compared to the EB assay suggests that complex **2c** exhibits a stronger preference for minor groove binding over interactive interaction.

### 2.11 Molecular docking study

The inhibition of the AR plays a crucial role in the treatment of androgen-dependent diseases, particularly prostate cancer. Targeting AR with small molecules can modulate its activity, thereby disrupting androgen signaling pathways critical for tumor growth and progression. In this context, our molecular docking study aimed to evaluate the binding affinity of Pd-based complexes as potential AR inhibitors. As shown in Table 6, molecular docking results indicate that the **2c** complex exhibits the strongest interaction with the receptor, as evidenced by its lowest binding free energy value. Its inhibition constant, the smallest among the tested compounds, suggests a high potential for enzyme inhibition, making it the most promising candidate in this study. The **2b** complex also demonstrates significant inhibitory activity, albeit slightly weaker than **2c**, yet it still forms stable interactions with the receptor, highlighting its relevance as a potential inhibitor. In contrast, the **2a** complex shows the highest binding free energy value and the largest inhibition constant, indicating a weaker affinity for the enzyme’s active site. For comparison, bicalutamide (**BIC**) was used as a reference inhibitor, and the results reveal that **2c** exhibits a stronger receptor binding affinity. These findings underscore the potential of **Pd** complexes as **AR** receptor inhibitors, with **2c** emerging as the most promising candidate for further investigation.

**TABLE 6:** Thermodynamic parameters (kcal/mol) of **Pd** complexes at the active site of Androgen Receptor **(AR)** determined after molecular docking simulation.

Complex	ΔG_*bind*_	K_i_ (µM)	ΔG_*inter*_	ΔG_*vdw + hbond + desolv*_	ΔG_*elec*_	ΔG_*total*_	ΔG_*tor*_	ΔG_*unb*_
AR								
2a	−5.56	83.36	−7.21	−7.23	0.02	−1.29	1.65	−1.29
2b	−8.44	0.65	−10.64	−10.62	−0.02	−1.74	2.20	−1.74
2c	−10.51	0.02	−12.16	−12.21	0.05	−2.47	1.65	−2.47
BIC	−9.11	0.21	−10.76	−10.65	−0.11	−1.92	1.65	−1.92

**FIGURE 12:**
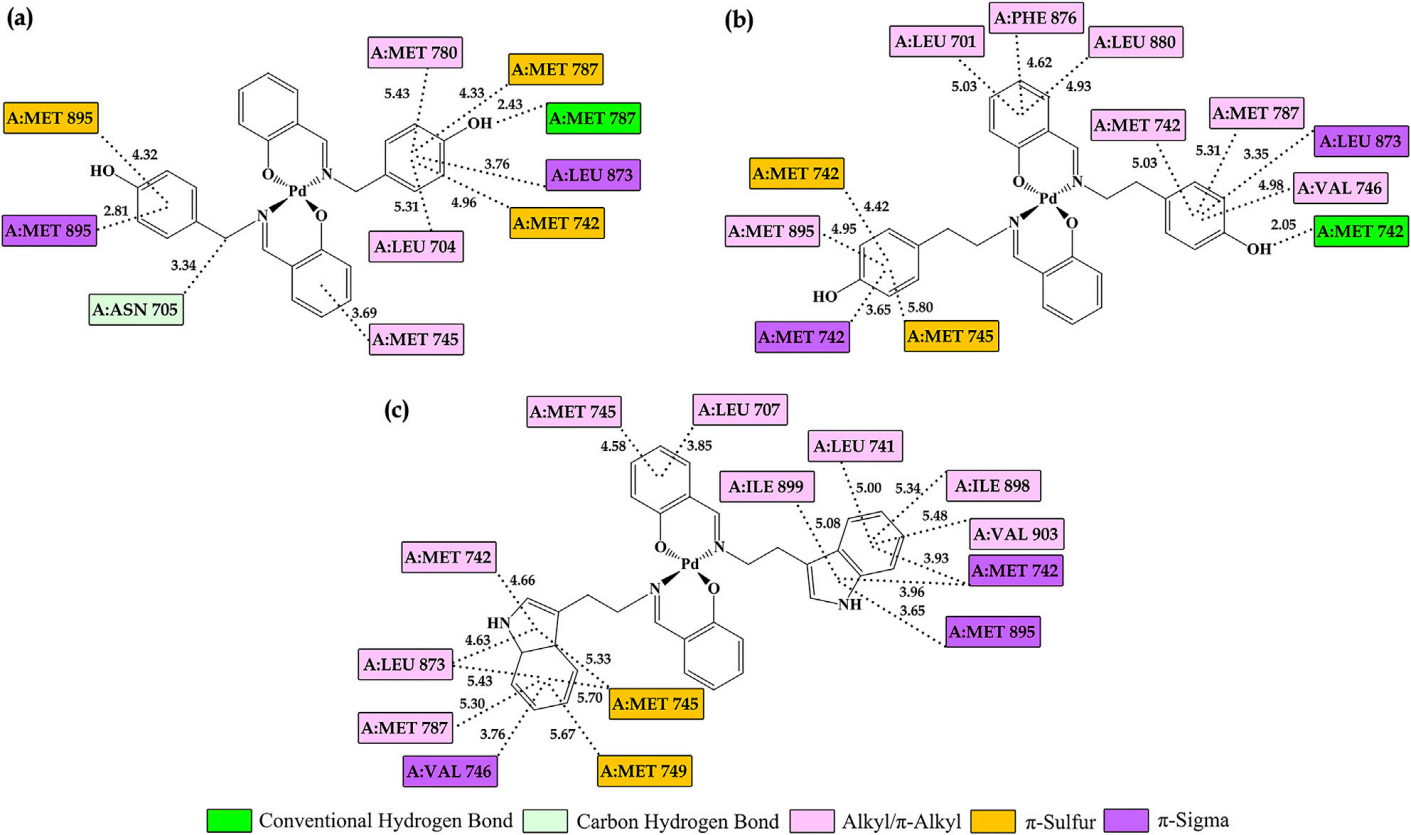
Visualization of key interactions between the Androgen Receptor **(AR)** and **(a) 2a**, **(b) 2b**, and **(c) 2c** in a 2D representation. Interatomic distances are measured in Å, with different colors indicating various interaction types, as described in the legend.

The molecular interactions between Pd derivatives and **AR**, illustrated in Figure 12, offer valuable insights into the potential inhibitory properties of these compounds. Non-covalent interactions contribute significantly to the stability of the ligand-protein complex by reinforcing the binding affinity within the enzyme’s active site. Hydrophobic interactions predominantly enhance the stability of the complex by facilitating favorable packing and minimizing solvent exposure, while conventional hydrogen bonds provide additional stabilization through directional electrostatic forces, further strengthening the ligand-enzyme interaction.

The hydrophobic segments of palladium derivatives enable interactions with various amino acids, such as A:LEU 704, A:VAL 746, and A:ILE 899, leading to the formation of alkyl/π-alkyl contacts. Additionally, other types of non-covalent interactions, including π-sigma and π-sulfur interactions, play a crucial role in ligand stabilization. π-sigma interactions arise due to the interaction between the electron cloud of aromatic rings and sigma bonds within the molecule (Ferreira de Freitas and Schapira, 2017), as observed in **2c** interacting with A:MET 742, A:VAL 746, and A:MET 895. Similarly, **2a** and **2b** also establish interactions with these amino acids, in addition to A:LEU 873. π-sulfur interactions, which involve interactions between sulfur atoms and the π-electron systems of aromatic rings (Meyer et al., 2003), are characteristic of enzymes containing methionine residues, including A:MET 742, A:MET 745, A:MET 749, A:MET 787, and A:MET 895.

Conventional hydrogen bonds are essential for maintaining the stability of ligand–enzyme complexes (Ferreira de Freitas and Schapira, 2017; Bissantz et al., 2010). These interactions arise between the hydroxyl groups of **2a** and **2b** derivatives and polar amino acid residues within the enzyme’s active site, such as A:MET 742 and A:MET 787. Moreover, a distinct carbon-hydrogen bond is formed between the **2a** derivative and A:ASN 705, further stabilizing the complex and ensuring the proper alignment of the ligand within the catalytic pocket. All these interactions collectively play a crucial role in stabilizing the ligand within the enzyme complex.

The observed interactions with the androgen receptor (**AR**) suggest that **Pd** complexes can effectively modulate receptor activity. Overall, these diverse binding modes highlight the potential of palladium derivatives for therapeutic applications, particularly in targeting key regulatory proteins like **AR**. The molecular docking results indicate that all examined Pd complexes exhibit significant interactions with key regulators of apoptosis–caspase 3 (**CASP3**), Bcl-2-associated X protein (**BAX**), and the anti-apoptotic B-cell lymphoma 2 (**BCL2**) protein. The negative values of the binding free energy (ΔG_bind_) suggest the stability of the formed complexes, while variations in the obtained parameters provide insight into the binding preferences of these compounds for pro-apoptotic (**CASP3**, **BAX**) and anti-apoptotic (**BCL2**) proteins.

Analysis of the data in Table 7 highlights **2c** as the strongest binding ligand, exhibiting the lowest ΔG_bind_ values for **CASP3** (−8.81 kcal/mol) and **BAX** (−8.44 kcal/mol). These results suggest that **Pd3** preferentially targets pro-apoptotic proteins, which may indicate its potential to induce cell death by activating apoptosis. These findings suggest that **2c** exhibits selectivity toward pro-apoptotic proteins, highlighting its potential role as a pro-apoptotic modulator in anticancer strategies. In contrast, **2a** and **2b** display a more balanced binding affinity between pro-apoptotic and anti-apoptotic proteins, which may imply a different mechanism of action.

**TABLE 7:** Thermodynamic parameters (kcal/mol) of **Pd** complexes at the active sites of caspase 3 **(CASP3)**, Bcl-2-associated X protein **(BCL2)**, and anti-apoptotic B-cell lymphoma 2 **(BAX)** determined after molecular docking simulations.

Complex	ΔG_*bind*_	Ki (µM)	ΔG_*inter*_	ΔG_*vdw + hbond + desolv*_	ΔG_*elec*_	ΔG_*total*_	ΔG_*tor*_	ΔG_*unb*_
2a								
CASP3	−7.88	1.66	−9.53	−9.49	−0.04	−1.18	1.65	−1.18
BCL2	−7.92	1.56	−9.57	−9.39	−0.18	−1.39	1.65	−1.39
BAX	−7.68	2.36	−9.32	−9.27	−0.05	−1.54	1.65	−1.54
2b								
CASP3	−7.98	1.42	−10.17	−9.92	−0.25	−1.90	2.20	−1.90
BCL2	−8.08	1.20	−10.27	−10.22	−0.06	−1.87	2.20	−1.87
BAX	−6.86	9.31	−9.06	−8.98	−0.08	−2.18	2.20	−2.18
2c								
CASP3	−8.81	0.35	−10.46	−10.41	−0.05	−2.89	1.65	−2.89
BCL2	−8.26	0.88	−9.91	−9.85	−0.06	−2.91	1.65	−2.91
BAX	−8.44	0.65	−10.09	−10.11	0.02	−2.72	1.65	−2.72

**FIGURE 13:**
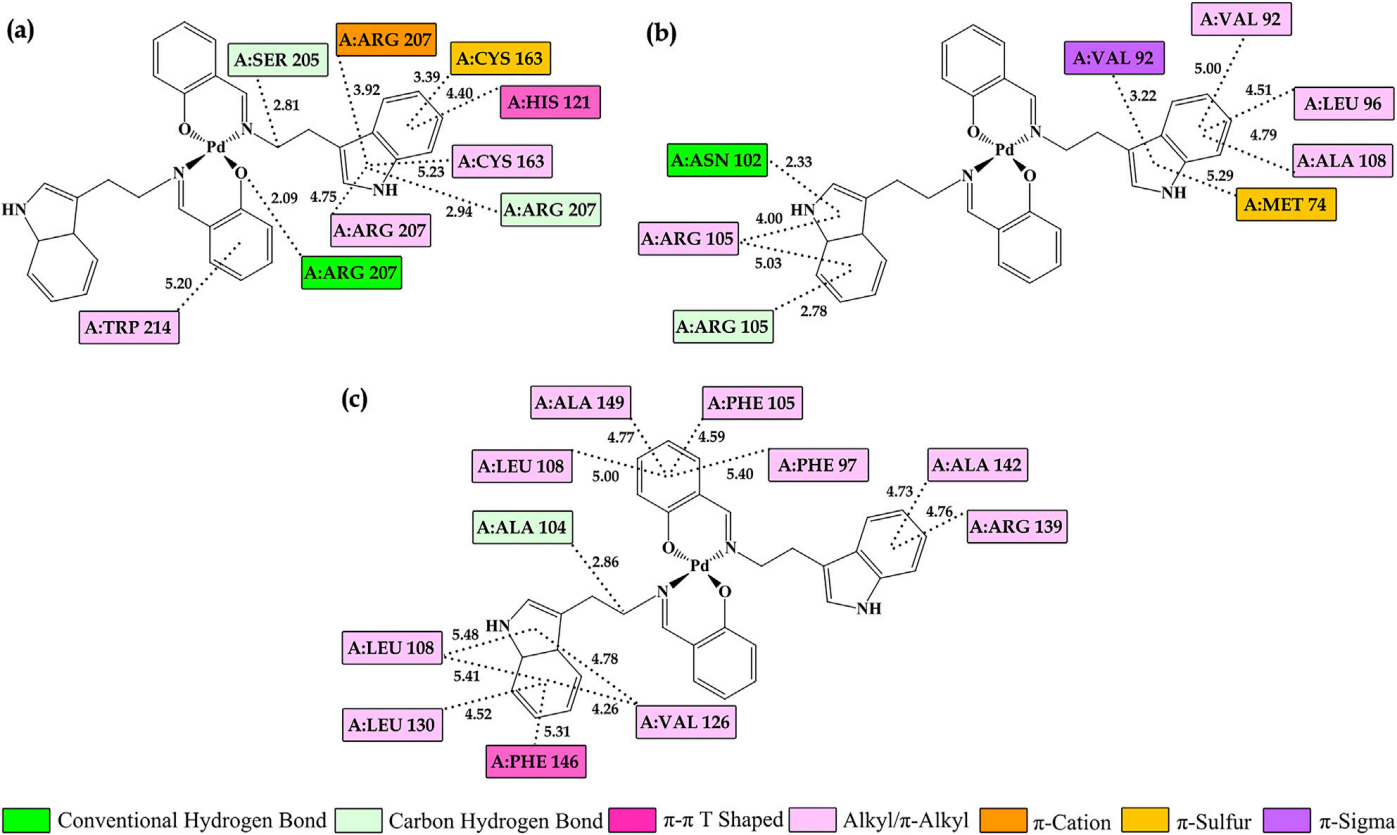
Visualization of key interactions between the **2c** and **(a)** caspase 3 **(CASP3)**, **(b)** Bcl-2-associated X protein **(BCL2)**, and **(c)** anti-apoptotic B-cell lymphoma 2 **(BAX)** in a 2D representation. Interatomic distances are measured in Å, with different colors indicating various interaction types, as described in the legend.

Since **2c** exhibited the most favorable binding results, it was selected to represent the intermolecular interactions with the active sites of **CASP3**, **BCL2**, and **BAX**, as shown in Figure 13. These interactions involve a combination of hydrophobic, electrostatic, and hydrogen bonding forces. Hydrophobic interactions play a central role in driving the binding process, while electrostatic forces aid in the precise alignment of **2c** within the enzyme’s active site. Additionally, hydrogen bonds further stabilize the complex, enhancing both the strength and specificity of the binding. These findings underscore the molecular factors that contribute to **2c**’s potential as an effective modulator of apoptotic pathways.

In the context of **CASP3** (Figure 13a), **2c** engages in a conventional hydrogen bond with A:ARG 207, along with carbon-hydrogen interactions involving A:SER 205 and A:ARG 207. Additionally, alkyl/π-alkyl interactions are observed with A:CYS 163, A:ARG 207, and A:TRP 214. The π-π interactions can adopt different configurations, such as edge-to-face (T-shape) or offset stacking (shifted parallel alignment), depending on the local geometry and chemical environment of the binding site (Meyer et al., 2003; Tsuzuki et al., 2002). Notably, A:HIS 121 participates in one of these π-π interactions, adopting an edge-to-face (T-shape) configuration. **2c** also establishes a π-sulfur interaction with A:CYS 163, further stabilizing its binding. Moreover, electrostatic interactions play a critical role in ligand stabilization, particularly π-anion and π-cation interactions, which arise when the electron cloud of an aromatic ring interacts with an anion or cation (Ferreira de Freitas and Schapira, 2017; Mahadevi and Sastry, 2013). In this context, **2c** forms a π-cation interaction with A:ARG 207, underscoring the selectivity of its binding. Together, these diverse interaction types contribute to a strong binding affinity, aligning with the observed activation of caspase-3. More broadly, they are essential for the stability and specificity of protein-ligand complexes, as they enhance charge complementarity between the ligand and active-site residues, ultimately strengthening overall binding affinity.

For **BCL2** (Figure 13b), **2c** establishes a conventional hydrogen bond with A:ASN 102 and a carbon-hydrogen bond with A:ARG 105. Additionally, alkyl/π-alkyl interactions are observed with A:VAL 92, A:LEU 96, A:ARG 105, and A:ALA 108, while π-sulfur hydrophobic interactions occur with A:MET 74. Notably, characteristic π-sigma interactions are also present with A:VAL 92. The inhibition of **BCL2** disrupts mitochondrial membrane stability, triggering the release of pro-apoptotic factors such as cytochrome c, a key molecule in the initiation of apoptosis.

In the case of **BAX** (Figure 13c), **2c** establishes a carbon-hydrogen bond with A:ALA 104 and engages in T-shaped π-π interactions with A:PHE 146. Additionally, alkyl/π-alkyl interactions are observed with multiple residues, including A:PHE 97, A:VAL 126, A:LEU 130, and A:ALA 149. These stabilizing interactions enhance **BAX** activation, promoting pore formation in the mitochondrial membrane, which facilitates the release of apoptotic factors and drives the process of programmed cell death.

The molecular interactions of **Pd**-based complexes with **CASP3**, **BCL2**, and **BAX** offer valuable insights into their potential role in modulating apoptotic pathways. Through diverse binding modes–including hydrogen bonding, π-π stacking, alkyl/π-alkyl interactions, π-sulfur interactions, and electrostatic forces–these complexes contribute to the stability and specificity of protein-ligand interactions. Collectively, these findings underscore the ability of **Pd**-based complexes to regulate key apoptotic proteins, highlighting their potential as therapeutic agents for apoptosis-related diseases. The specificity and strength of their interactions with critical residues further reinforce their significance in fine-tuning apoptotic signaling pathways.

The integrated results of cytotoxicity assays, DNA interaction studies, and molecular docking analyses demonstrate that Pd(II) complex **2c** exhibits the most potent and selective anticancer activity among the tested compounds. Complex **2c** reduced the viability of DU-145 and PC-3 prostate cancer cells more effectively than cisplatin at later time points while showing lower toxicity toward non-cancerous fibroblasts, suggesting improved therapeutic selectivity. Mechanistically, **2c** triggered apoptosis via the intrinsic mitochondrial pathway, as evidenced by Bax upregulation, Bcl-2 downregulation, caspase-3 activation, mitochondrial membrane potential loss, and G0/G1 cell cycle arrest. Fluorescence displacement assays confirmed that **2c** interacts with DNA via both intercalation and minor groove binding, with a preference for the latter. Molecular docking supported these findings, revealing strong and specific binding of **2c** to pro-apoptotic proteins (caspase-3, Bax), with moderate affinity for anti-apoptotic Bcl-2. Although AR-negative cell lines were used in this study, complex **2c** also showed a high predicted binding affinity for the androgen receptor, suggesting potential applicability in AR-positive prostate cancer models. These combined results suggest that complex **2c** exerts its cytotoxic effects through a multi-targeted mechanism involving DNA engagement, apoptosis induction, and modulation of key signaling proteins, highlighting its promise as a candidate for further development in prostate cancer therapy.

## 3 Materials and Methods

### 3.1 General procedures

#### 3.1.1 General procedures and materials

All chemicals were used without purification, and all of them are commercially available. ^1^H NMR and ^13^C NMR spectra were recorded in DMSO-d_6_ as a solvent with a Bruker Ascend 400 (400 MHz) spectrometer. Chemical shifts are given in parts per million (δ) down from tetramethylsilane as the internal standard. For elemental analyses (C, H, O, N) were used Elementar Vario MICRO elemental analyzer. IR spectra were obtained on Perkin Elmer FT-IR spectrometer, two equipped with a DTGS detector. Mass spectra were recorded at 6546 LC/Q-TOF (Agilent, Santa Clara, CA, SAD) equipped with Jet Stream ion source and connected with 1,290 Infinity II HPLC (Agilent, Santa Clara, CA, AD). Data were collected every second in the mass area 100–1,000 Da. Molar conductance of the Pd(II) complexes was measured on freshly prepared 10^–3^ M solutions in DMSO at room temperature using Crison EC-Meter basic 30+conductivity cell. The viscosity of the DNA solution was measured in the absence and presence of increasing amounts of **2c**. To the 0.01 mM DNA solution in PBS, a corresponding ratio of examinated compound was added at ratio [**2c**]/[DNA]: 0.1; 0.2; 0.3; 0.4; 0.5; 0.6; 0.7; 0.8; 0.9 and 1. Data are presented as a plot of (η/η0)1/3 vs. ratio of [**2c**]/[DNA], where η is the DNA viscosity in the presence of the compound, η0 is the viscosity of DNA in buffer alone.

#### 3.1.2 General procedure for the synthesis of ligand compounds 1a–1c

15 mL of absolute ethanol and 1 mmol of the corresponding amine (*p*-OH benzyl amine for **1a**, tyramine for **1b**, and tryptamine for **1c**) were added to a 50 mL flask. After dissolving the amine, an equal amount of salicylic aldehyde was added, and the reaction mixture was stirred at room temperature overnight. The obtained solid products were filtered, washed with cold ethanol, and dried in an air atmosphere. The spectral data for the compounds were in agreement with those previously published (Babić et al., 2025; Marjanović et al., 2024). The corresponding spectra of all ligands compounds are given in Supplementary Material.

#### 3.1.3 Experimental procedure for the synthesis of palladium complexes 2a–2c

0.0001 mol of the corresponding Schiff base ligand (**1a–1c**) was dissolved in 5 mL of acetonitrile and subsequently equivalent of K_2_PdCl_4_ was added in one portion. The resulted mixture was stirred at room temperature for 4 h. The obtained solid products were filtered, washed thoroughly with ethanol and dried under air atmosphere. The obtained palladium complexes **2a–2c** were characterized by ^1^H and ^13^C NMR, IR, MS spectra and elemental analysis.

**2a**: Yellow powder. Yield 84%. ^1^H NMR (400 MHz, DMSO-d_6_) δ 9.35 (s, 2H, *p*-OH-benzylamine-O-H), 8.20 (s, 2H, -N=C-H), 7.56–7.05 (m, 4H, from salicylaldehyde-Ar-H and 4H from *p*-OH-benzylamine-Ar-H), 6.93–6.47 (m, 8H, 4H from salicylaldehyde-Ar-H and 4H from *p*-OH-benzylamine-Ar-H), 4.82 (s, 4H, -CH2-).^13^C NMR (101 MHz, DMSO-d_6_) δ 163.69, 163.26, 157.68, 156.56, 134.66, 130.47, 129.56, 129.12, 124.04, 120.30, 119.68, 115.21, 114.93, 57.41. IR (cm^-1^): 1,615 ν(C=N), 513 ν(Pd−N), 461 ν(Pd−O).Anal. Found: C, 60.28; H, 4.41; N, 5.12. Calc. for C_28_H_24_N_2_O_4_Pd: C, 60.17; H, 4.33; N, 5.01.

**2b**: Orange powder. Yield 75%. ^1^H NMR (400 MHz, DMSO-*d*_6_) δ 9.21 (s, 2H, tyramine-OH), 7.88 (s, 2H, -N=C-H), 7.27 (dd, J = 7.5, 5.7 Hz, 4H from tyramine-ArH and salicilal. ArH), 7.10 (d, J = 8.4 Hz, 4H, *p*-OH-benzylamine-Ar-H), 6.82–6.52 (m, 8H, 4H, salicilal.-ArH and tyramine-Ar-H), 3.87 (t, J = 7.4 Hz, 4H -CH_2_-), 2.91 (t, J = 7.5 Hz, 4H -CH_2_-). ^13^C NMR (101 MHz, DMSO*-d*_6_) δ 163.54 (-N=C-), 163.06, 155.72, 134.55, 129.83, 128.90, 120.21, 119.59, 115.19, 114.82, 58.44, 37.42. IR (cm^-1^): 1,611 ν(C=N), 506 ν(Pd−N), 460 ν(Pd−O). Anal. Found: C, 61.49; H, 4.74; N, 4.85. Calc. for C_30_H_28_N_2_O_4_Pd: C, 61.39; H, 4.81; N, 4.77.

**2c**: Dark red-brown powder. Yield 78%. ^1^H NMR (400 MHz, DMSO-d_6_) δ 10.96 (s, 2H, -N-H), 7.96 (s, 2H, -N=C-H), 7.6–6.90 (m, 2H, tryptamine-Ar-H; m, 6H, 4H from salicyal.-Ar-H, 2H from tryptamine Ar-H; 2H, tryptamine-Ar-H and 2H, tryptamine-Ar-H, 2H, tryptamine-Ar-H; 4H, salicylal.-Ar-H), second triplet from the CH_2_ group overlapped with the H_2_O peak, 3.02 (t, J = 8.0 Hz, 4H, -CH_2_-). ^13^C NMR (101 MHz, DMSO-d_6_) δ 164.06 (-N=C-), 163.34, 136.76, 136.66, 129.73, 127.24, 123.83, 121.66, 118.97, 118.54, 117.70, 115.24, 112.02, 111.68, 57.85, 28.51. IR (cm^-1^): 1,618 ν(C=N), 559 ν(Pd−N), 453 ν(Pd−O). Anal. Found: C, 64.38; H, 4.72 N 8.79. Calc. for C_34_H_30_N_4_O_2_Pd: C, 64.51; H, 4.78; N, 8.85.

### 3.2 *In vitro* cytotoxic study

#### 3.2.1 Cell cultures

In this investigation, two experimental groups of cancer cell lines were employed: human prostate carcinoma cells lacking prostate-specific membrane antigen (PSMA) and androgen receptor independence (DU-145 and HTB-81™), alongside human prostate carcinoma cells (PC-3 and CRL-1435™). The control group comprised non-cancerous human fibroblasts (MRC-5 and CCL-171™). All cell lines utilized in this study were procured from the American Type Culture Collection (ATCC, Manassas, VA, United States). The cells were maintained in a complete medium containing high-glucose DMEM, supplemented with 10% fetal bovine serum and 200 mM L-glutamine (reagents sourced from Sigma-Aldrich, St. Louis, MO, United States). Cultivation of the cells was conducted in 25 cm^2^ flasks (Thermo Fisher Scientific, Waltham, MA, United States) at 37°C under conditions of absolute humidity and a 5% CO_2_ atmosphere.

#### 3.2.2 MTT assay

The cytotoxic effects of three newly synthesized Pd(II) complexes were assessed on DU-145, PC-3, and MRC-5 cell lines using the MTT assay. For comparison, the cytotoxicity of cisplatin, a clinically established chemotherapeutic agent, was also evaluated across all cell lines. Cells were harvested during their exponential growth phase, counted, and seeded at a density of 5 × 10^3^ cells per well in 96-well culture plates. Following an initial 24-h incubation at 37°C in a humidified atmosphere with 5% CO_2_, the cells were treated with varying concentrations of Pd(II) complexes and cisplatin (0.3, 1, 3, 10, 30, and 100 μM), alongside a control group maintained in complete medium. The treated cells were incubated under identical conditions (37°C, 5% CO_2_, absolute humidity) for durations of 24, 48, and 72 h. After incubation, the medium was removed, and an MTT solution was added to each well. The cells were then incubated for an additional 2 h to facilitate the reduction of thiazolyl blue tetrazolium bromide in the MTT solution. Subsequently, the MTT solution was carefully removed, and the resulting formazan crystals were solubilized in DMSO. The plates were shaken in the dark for 10 min before measuring the absorbance of the purple-colored solution at 595 nm using a microplate reader (Zenyth 3,100, Anthos Labtec Instruments, Salzburg, Austria). All experiments were performed in triplicate and repeated across three independent runs. Cell viability was calculated as a percentage by dividing the absorbance of treated cells (minus blank absorbance) by the average absorbance of untreated control cells (minus blank absorbance) and multiplying by 100.

$$\%\text{ of the viable cells}=\left(\left(\text{absorbance of treated cell}-\text{absorbance of blank}\right)\;/\;\left(\text{absorbance of untreated cell}-\text{absorbance of blank}\right)\right)\;*\;100$$

The IC_50_ values, representing the concentration required to reduce cell viability by 50% relative to the control, were calculated by fitting the logarithmically transformed dose-response data obtained from the MTT assay. This analysis was performed using Microsoft Office Excel 2010, leveraging its curve-fitting capabilities to determine precise inhibitory concentrations.

#### 3.2.3 Annexin V/7AAD assay

The mode of cell death triggered by Pd(II) complexes **2a–2c** was assessed using the Annexin V–fluorescein isothiocyanate (FITC)/propidium iodide (PI) Apoptosis Kit (BD Biosciences, Franklin Lakes, NJ, United States). DU-145 cells were exposed to the respective IC_50_ concentrations of the **2a–2c** complexes or cultured in media alone as a control for 24 h at 37°C in a humidified atmosphere with 5% CO_2_. Following incubation, the cells were trypsinized, washed with phosphate-buffered saline (PBS), centrifuged, and resuspended in 100 μL of ice-cold binding buffer. Subsequently, the cells were stained with 10 μL of Annexin V-FITC and 20 μL of PI, incubated for 15 min at room temperature in the dark, and then supplemented with 400 μL of binding buffer per tube. Flow cytometric data were analyzed using a standardized gating strategy to ensure accurate identification of cell populations. Initially, cell debris and doublets were excluded based on forward and side scatter properties. Subsequent gating was performed on singlet populations to minimize aggregation artifacts. For apoptosis assessment using Annexin V-FITC and propidium iodide (PI), compensation was applied using single-stained controls to correct for spectral overlap. Unstained and untreated control samples were used to establish baseline fluorescence and define quadrant boundaries. Cells were then categorized into four populations: viable (Annexin V−/PI−), early apoptotic (Annexin V+/PI−), late apoptotic or secondary necrotic (Annexin V+/PI+), and necrotic (Annexin V−/PI+). A minimum of 10,000 events per sample were recorded, and all gates were applied consistently. The samples were analyzed using a Cytomics FC500 flow cytometer (Beckman Coulter, Brea, CA, United States), and the data were processed with FlowJo V10 Software. The results were presented as density plots illustrating Annexin V-FITC and PI staining patterns.

#### 3.2.4 Cell cycle

The next phase of the research involved investigating the effects of Pd(II) complexes **2a–2c** on the cell cycle progression of DU-145 cells. DU-145 cells were treated with IC_50_ concentrations of Pd(II) complexes **2a–2c** and cisplatin, or cultured in media alone as a control, for 24 h at 37°C under conditions of 5% CO_2_ and absolute humidity. Following incubation, the cells were harvested, washed with phosphate-buffered saline (PBS), and fixed in 70% ethanol at +4°C. The fixed cells were aggregated and resuspended in 1 mL PBS containing RNAse A (500 μg/mL). After a 30-min incubation at 37°C, the cells were stained with 5 μL propidium iodide (PI) solution (10 mg/mL PBS). The samples were incubated for an additional 15 min in the dark and subsequently analyzed using a flow cytometer. Cell cycle distribution data were processed using FlowJo V10 Software and presented as histograms.

#### 3.2.5 Assessment of apoptosis

Our research focused on examining the expression levels of the pro-apoptotic protein Bax, the anti-apoptotic protein Bcl-2, and the percentage of cells containing active caspase-3. DU-145 cells were incubated for 24 h with the IC_50_ concentrations of Pd(II) complexes **2a–2c** or in complete cell culture medium as a control. Following incubation, the cells were washed three times with ice-cold PBS, resuspended, fixed, and permeabilized using a Fixation and Permeabilization Kit (eBioscience, San Diego, CA, United States). For Bcl-2 staining, cells were incubated with a 1:1,000 Bcl-2 fluorescein isothiocyanate (FITC) primary antibody (mhbcl01, Life Technologies, Thermo Fisher Scientific, Waltham, MA, United States) for 15 min at room temperature. Additional staining involved incubating permeabilized DU-145 cells for 30 min with 1:1,000 primary antibodies for active Bax (N20, sc-493; Santa Cruz Biotech Inc., Dallas, TX, United States) and cleaved caspase-3 (#9661, Cell Signaling Technology, Danvers, MA, United States). Cells were then washed with PBS and incubated with a 1:2000 secondary goat anti-rabbit IgG-FITC antibody (Ab6717-1, Abcam, Cambridge Biomedical Campus, Cambridge, United Kingdom) for 30 min. Subsequently, cells were washed in PBS and analyzed by flow cytometry. Fluorescence from at least 15,000 events per sample was measured using a Cytomics FC500 flow cytometer (Beckman Coulter, Brea, CA, United States). Fluorescence intensity was standardized using isotype-matched negative control antibodies. The mean fluorescence intensities (MFIs) for Bax and Bcl-2 were calculated as the ratio of raw mean channel fluorescence to isotype control levels, respectively, and represented the expression levels of these proteins. The concentrations of cleaved caspase-3 were evaluated as the percentages of cells displaying fluorescence.

#### 3.2.6 Clonogenic assay

In this assay (Franken et al., 2006), cells are seeded at low densities and incubated for 2 weeks to allow colony formation, with colonies defined as clusters of ≥50 cells. Treated and untreated cells are compared to assess survival fractions (SF), calculated by normalizing the plating efficiency (PE) of treated cells to that of controls. Colonies are fixed with 6% glutaraldehyde, stained with 0.5% crystal violet, and colonies were quantified using ImageJ software version 1.54 with a standardized macro. Images were first converted to 8-bit grayscale, and background noise was reduced using the “Subtract Background” function. Thresholding was applied to isolate colonies, and the “Analyze Particles” tool was used to count colonies based on defined size and circularity parameters. Only colonies with an area corresponding to ≥50 cells and a circularity range of 0.3–1.0 were included. Overlapping or merged colonies were excluded unless distinct borders were visually discernible. All image processing steps were applied consistently across samples to ensure reproducibility. Data are represented as dose-response survival curves.

Plating efficiency is calculated by the following formula:

$$PE=\frac{Number\;of\;colonies}{Number\;of\;cells\;seeded}\times 100\%$$

Survival fraction is calculated when all plating efficiencies are calculated according to the formula:

$$SF=\frac{{PE}_{treated}}{{PE}_{control}}$$

#### 3.2.7 Assessment of mitochondrial membrane potential by JC-10 immunofluorescence staining

JC-10 gathers in the mitochondria of living cells, forming red fluorescence aggregates. On the other hand, JC-10 is also present in a monomeric cytosolic form and dyes cells green in necrotic and apoptotic cells. Mitochondrial membrane potential was assessed using the dye JC-10. DU-145 cells were seeded at a density of 2 × 10^4^ cells per well in 24-well cell culture plates and incubated in complete DMEM medium at 37°C with 5% CO_2_ for 24 h. The cells were treated with palladium complexes **2a–2c** at their IC_50_ concentrations for a duration of 48 h. The cells were treated for 20 min with 2.5 μM JC-10 dye in warm PBS. JC-10 accumulation in cells was evaluated using a Gramma Libero FLUO500T trinocular inverted fluorescence microscope. We used the 525/590 nm fluorescence emission ratio for our quantification analysis. We captured images using the 2020 S-EYE Setup Microscope camera and the S-EYE_Setup-1.6.0.11 software. The ImageJ program was used to analyse the images.

### 3.3 DNA interaction study

The EB/Hoe-competitive studies of complex **2c** were made using fluorescence emission spectroscopy. DNA-EB and DNA-Hoe solutions were prepared by mixing 100 μM EB/Hoe and 40 µM CT-DNA in phosphate buffered saline buffer at pH = 7.4. The fluorescence spectra were recorded immediately after the addition of an increasing amounts of compound **2c** (0–100) into the solution of DNA-EB or DNA-Hoe to investigate the possible binding effect of tested compound. The excitation wavelength was 527 nm for EB and 346 nm for Hoe, respectively, while emission was recorded in the wavelengths ranges 550–750 nm for EB and 360–600 nm for Hoe.

### 3.4 Stability assay

The complex used for the testing was dissolved in the minimal amount of DMSO (dimethylsulfoxide) possible and diluted with PBS of ∼10^–5^ M in concentration and less than 0.7% DMSO. UV-Vis spectra were recorded after 0, 24 and 48 h in the range 200–600 nm. Absorption spectra were recorded on UV–Vis Perkin–Elmer Lambda 35 spectrophotometer equipped with water thermostated cell.

### 3.5 Computational study

A computational approach was employed to assess the binding affinity and inhibitory potential of **2a–2c** complexes toward androgen receptor (AR), caspase 3 (CASP3), B-cell lymphoma 2 (BCL2), and Bcl-2-associated X protein (BAX). The molecular structures of the studied complexes were initially optimized using the *Gaussian16* software package (Frisch et al., 2019), applying the B3LYP–D3BJ functional (Zhao et al., 2006). The 6–311+G (d,p) basis set was applied for C, N, O, and H atoms, while Pd atoms were treated with the def2-TZVPD basis set (Becke and Johnson, 2005) incorporating an effective core potential. This optimization ensured accurate geometric and electronic properties, providing a solid foundation for further computational studies. The crystallographic structures of the target proteins were retrieved from the RCSB Protein Data Bank: AR (PDB code: 1Z95) (Bohl et al., 2005), CASP3 (PDB code: 3KJF) (Wang et al., 2010), BCL2 (PDB code: 2W3L) (Porter et al., 2009), and BAX (PDB code: 2YXJ) (Lee et al., 2007). The preparation of protein structures was carried out using BIOVIA Discovery Studio 4.0 (BIOVIA, 2024), where non-essential heteroatoms, co-crystallized ligands, and water molecules were removed. This step ensured unobstructed access to the active site, creating optimal conditions for docking. Molecular docking simulations were performed using AutoDock Tools 1.5.7, integrated with AutoDock 4.2.6 (Morris et al., 1998). The Lamarckian Genetic Algorithm (LGA) (Fuhrmann et al., 2010) optimized ligand conformations within a rigid protein framework. To comprehensively encompass the binding regions, docking grids were defined for each target protein: AR (69 × 69 × 69 Å^3^, centered at 27.977 × 2.357 × 6.252 Å), CASP3 (56 × 56 × 56 Å^3^, centered at 21.577 × −5.202 × 10.866 Å), BCL2 (66 × 66 × 66 Å^3^, centered at 39.806 × 26.936 × −12.415 Å), and BAX (69 × 69 × 69 Å^3^, centered at −9.874 × −14.522 × 10.799 Å), with a uniform grid spacing of 0.375 Å. During the docking process, ligand flexibility was allowed, while the protein structure remained rigid, ensuring the identification of potential binding modes accurately. The LGA parameters were set to include a population size of 150, a maximum of 2,500,000 energy evaluations over 27,000 generations, a mutation rate of 0.02, and a crossover rate of 0.8. This approach enabled a comprehensive analysis of ligand-protein interactions, providing valuable insights into the inhibitory potential of Pd-based complexes.

## 4 Conclusion

Three new palladium complexes **2a–2c**, derived from Schiff base ligands combing salicylic and tyramine/tryptamine or *p*-hydroxyl benzylamine scaffolds in structure, have been synthesized and characterized. The complexes displayed potent anti-proliferative activity against both human prostate cancer cell lines PC-3 and DU-145 and good selectivity between cancer and normal cells. Complex **2c,** combining salicylic and tryptamine structural motifs, has demonstrated even better activity than cisplatin for both tested cell lines, with IC_50_ values of 7.1 µM (DU-145) and 8.6 µM (PC-3). The apoptotic assay has revealed the potential of complexes for apoptosis induction through the Bcl-2 and caspase family activation pathways. Molecular docking studies revealed that complex **2c** exhibits the strongest binding affinity toward the androgen receptor (AR), surpassing the reference inhibitor bicalutamide. Additionally, **2c** showed selective interactions with pro-apoptotic proteins **CASP3** and **BAX**, involving stable non-covalent forces such as hydrophobic contacts, π-π stacking, π-sulfur, and hydrogen bonds. These interactions suggest a dual mechanism of action–AR inhibition and pro-apoptotic modulation–supporting **2c**’s potential as a multifunctional anticancer agent. Taken together, these findings highlight the therapeutic potential of palladium complex **2c** as a promising lead compound for the development of new, multitarget anticancer agents, particularly in the treatment of prostate cancer. In conclusion, the synthesized palladium complexes demonstrated promising *in vitro* anticancer activity, indicating their potential as lead compounds for further development. However, a limitation of the present study is the absence of *in vivo* data, which is essential to fully assess the therapeutic potential and safety profile of these complexes under physiological conditions. Future research should focus on evaluating the efficacy and toxicity of these compounds in appropriate *in vivo* models to support their progression toward clinical relevance.

## Data Availability

The original contributions presented in the study are included in the article/Supplementary Material, further inquiries can be directed to the corresponding authors.
