# Supplementary material for: Evaluation of newly synthesized schiff base Pd(II) complexes for prostate cancer treatment through in vitro cytotoxicity and molecular mechanistic studies
Source: Front Chem. 2025 Jul 17;13:1636477. doi: 10.3389/fchem.2025.1636477 (PMC12310718; doi:10.3389/fchem.2025.1636477)

**Figure SI1.** ^1^H-NMR spectrum of **1a**


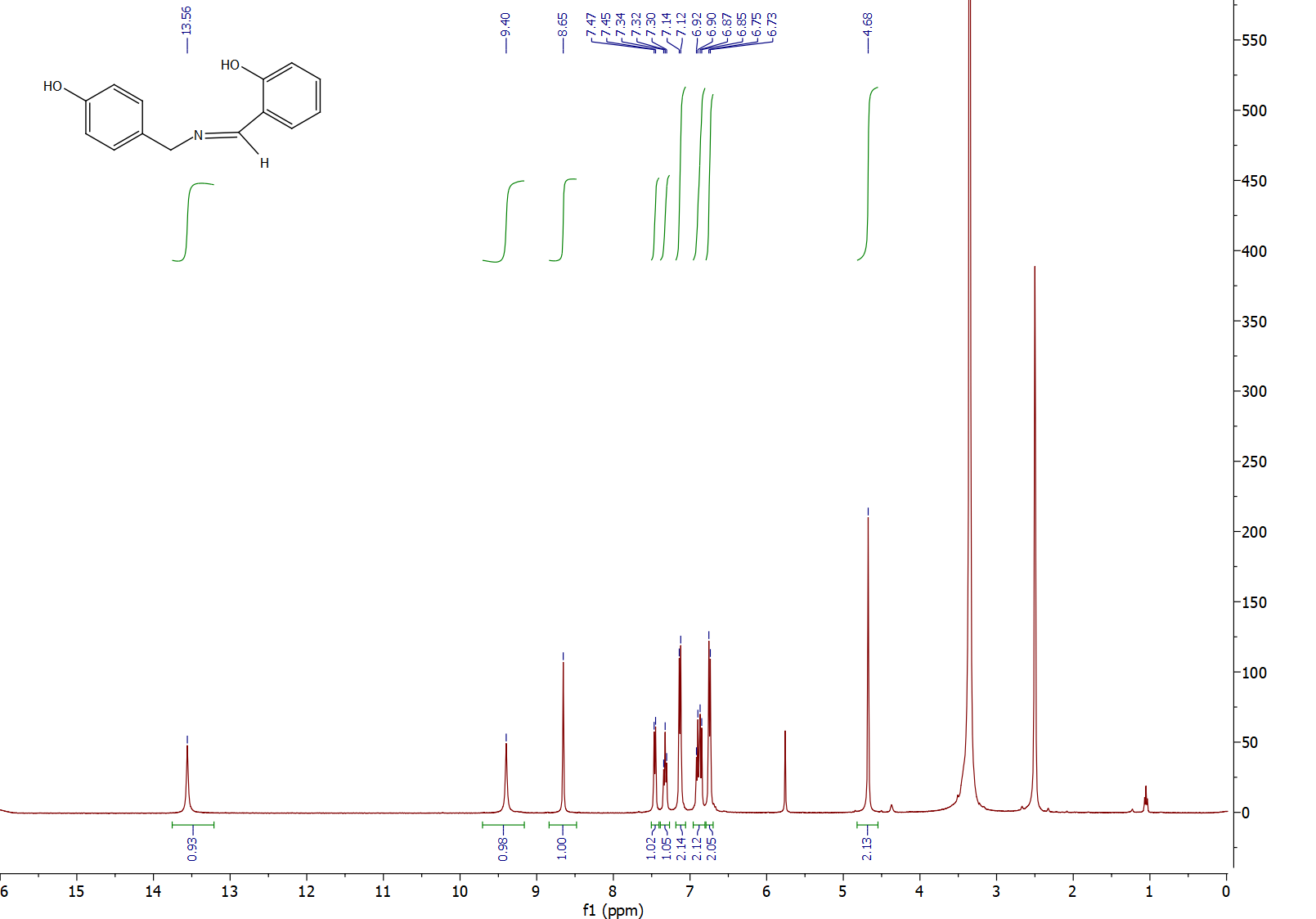


**Figure SI2.** ^13^C-NMR spectrum of **1a**


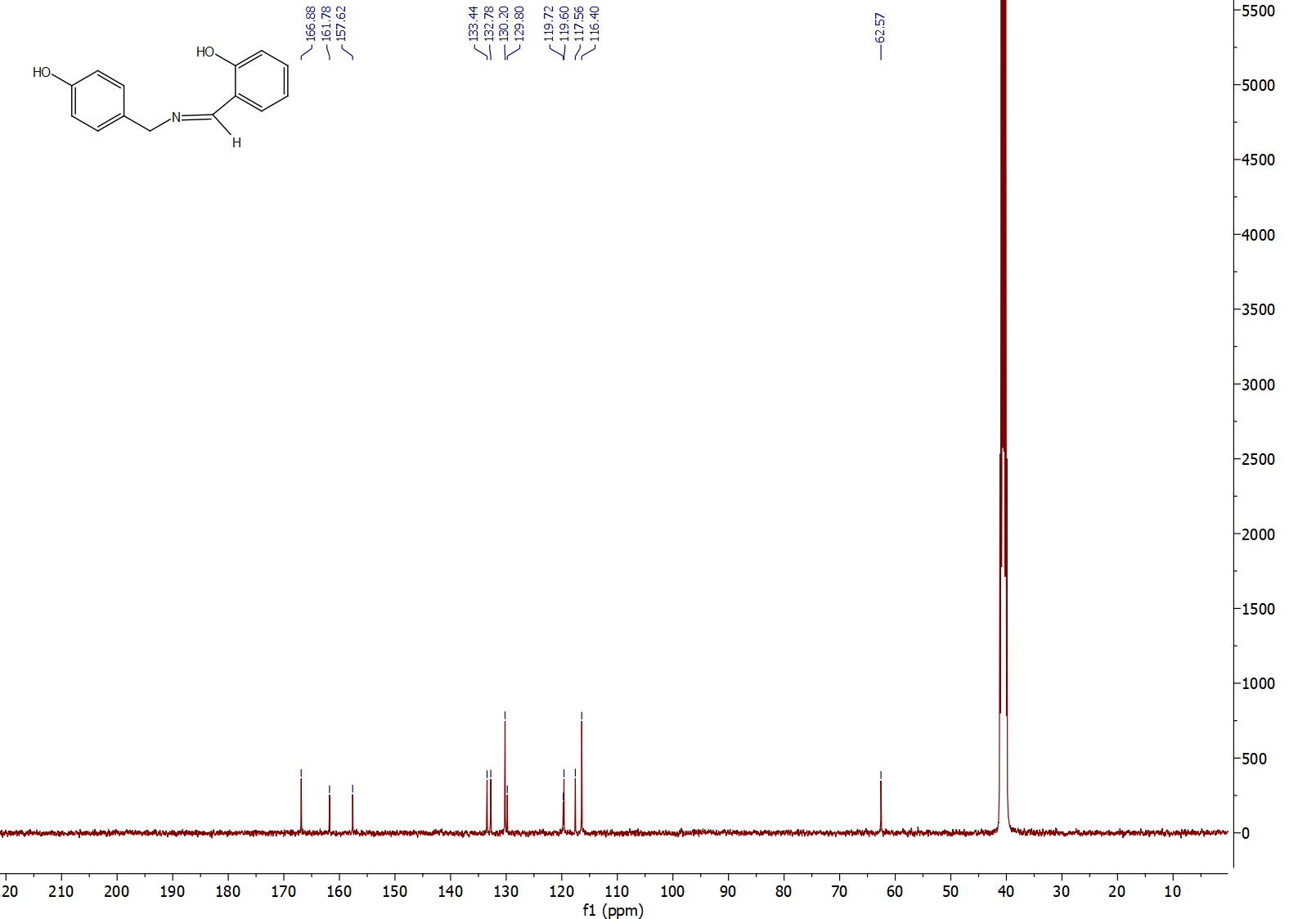


**Figure SI3.** IR spectrum of **1a**


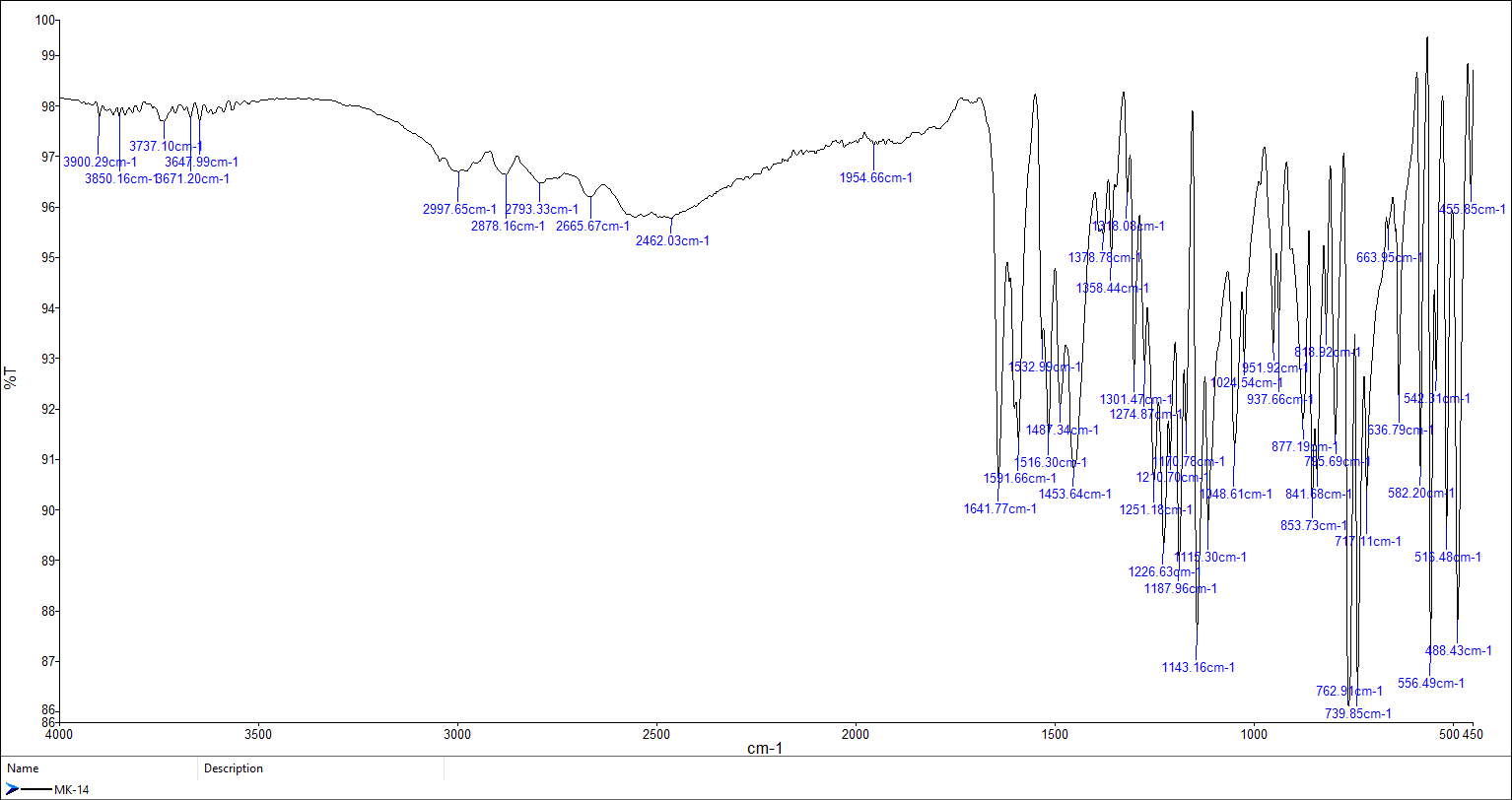


**Figure SI4**. ^1^H NMR spectra of compound **1b**


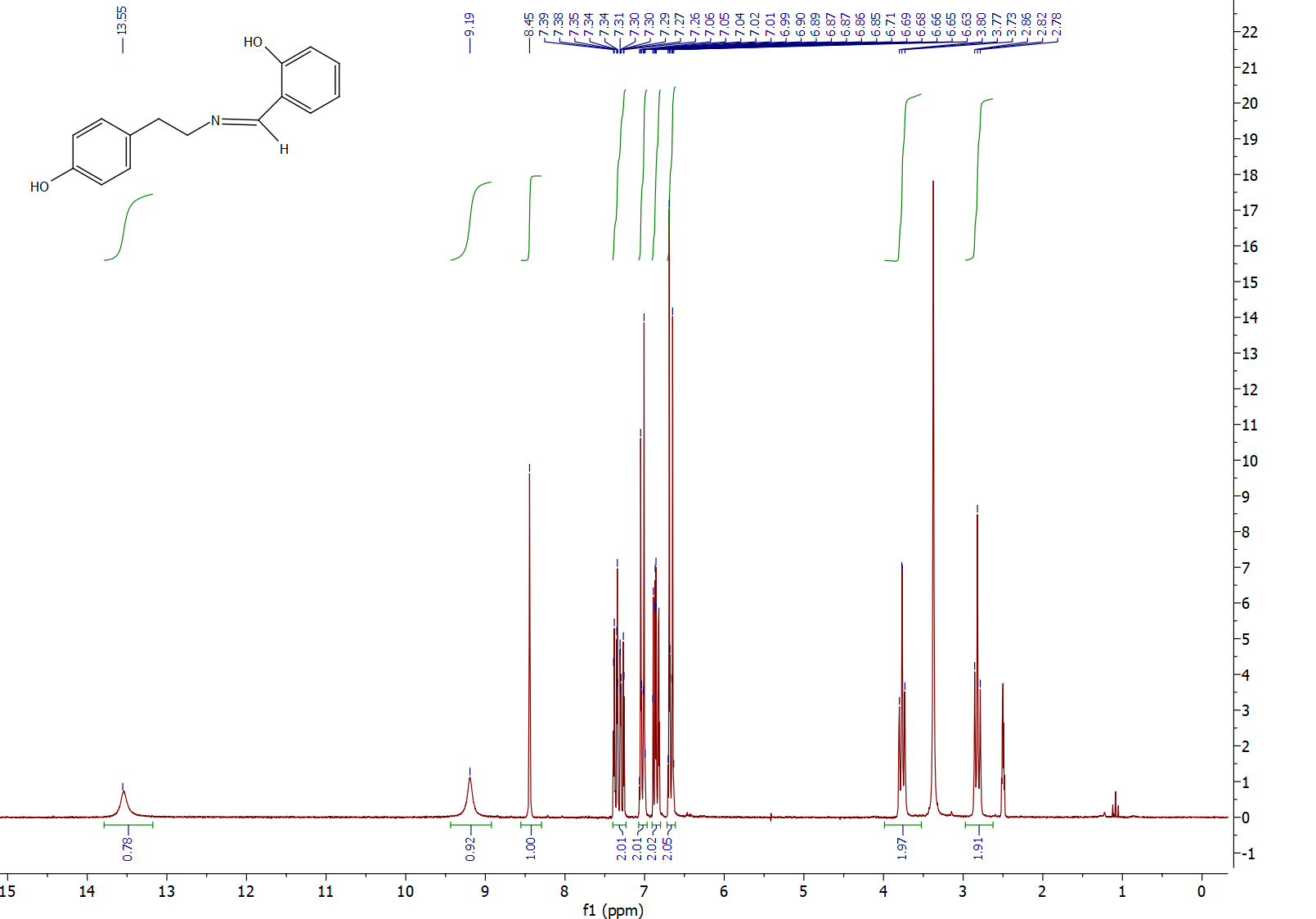


**Figure SI5**. ^13^C NMR spectra of compound **1b.**


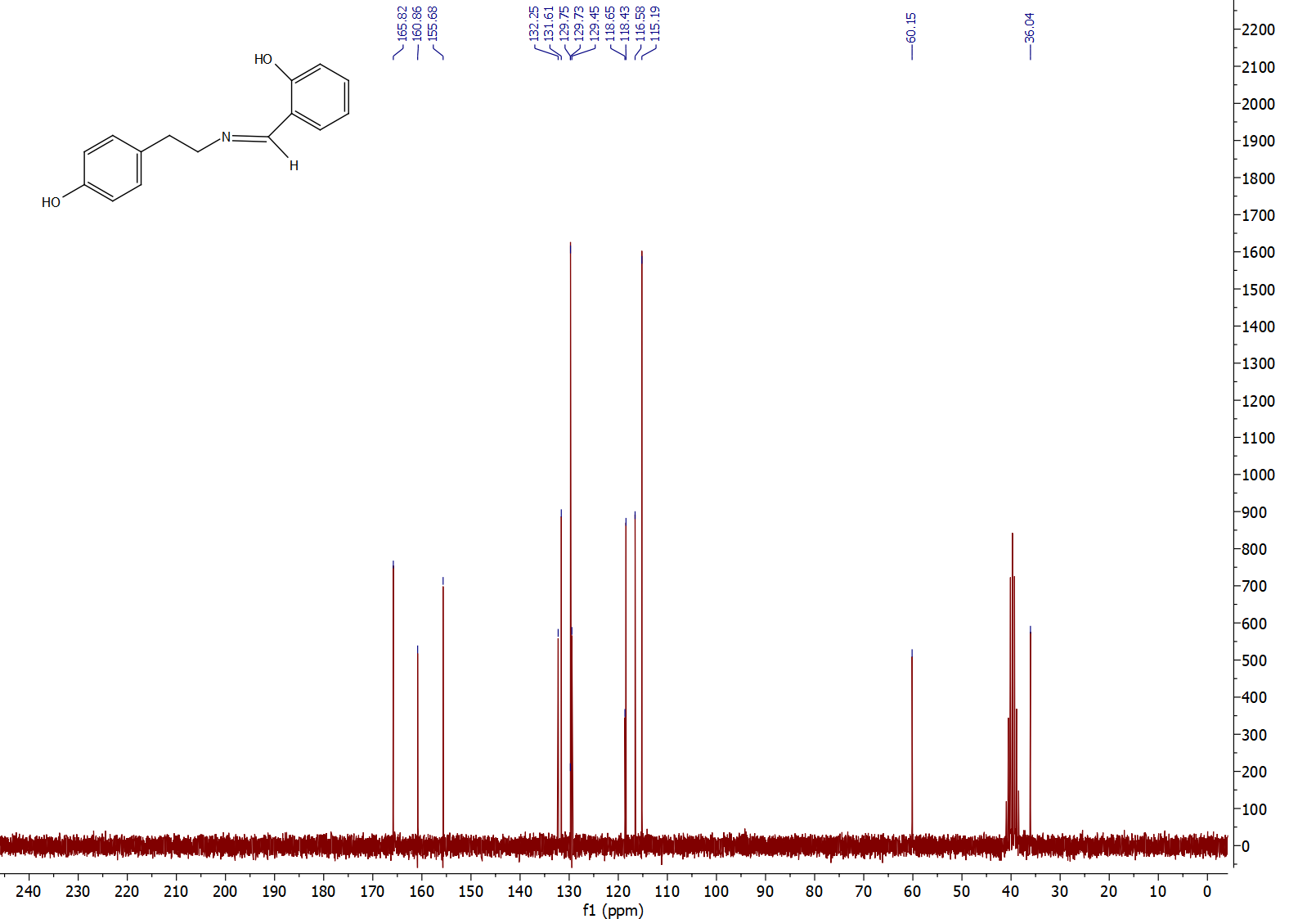


**Figure SI6**. IR spectra of compound **1b**.


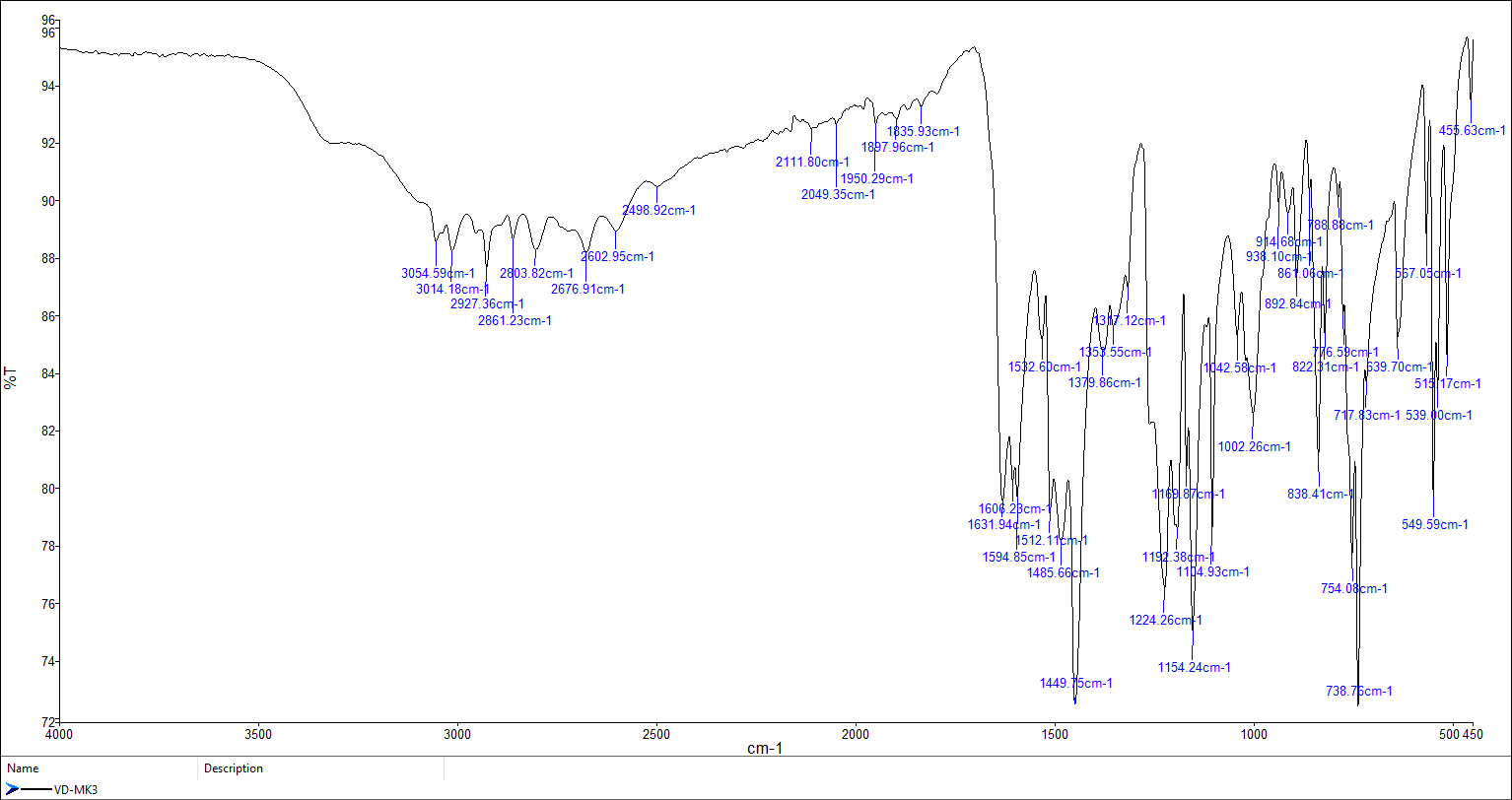


**Figure SI7.** ^1^H NMR spectra of compound **1c**


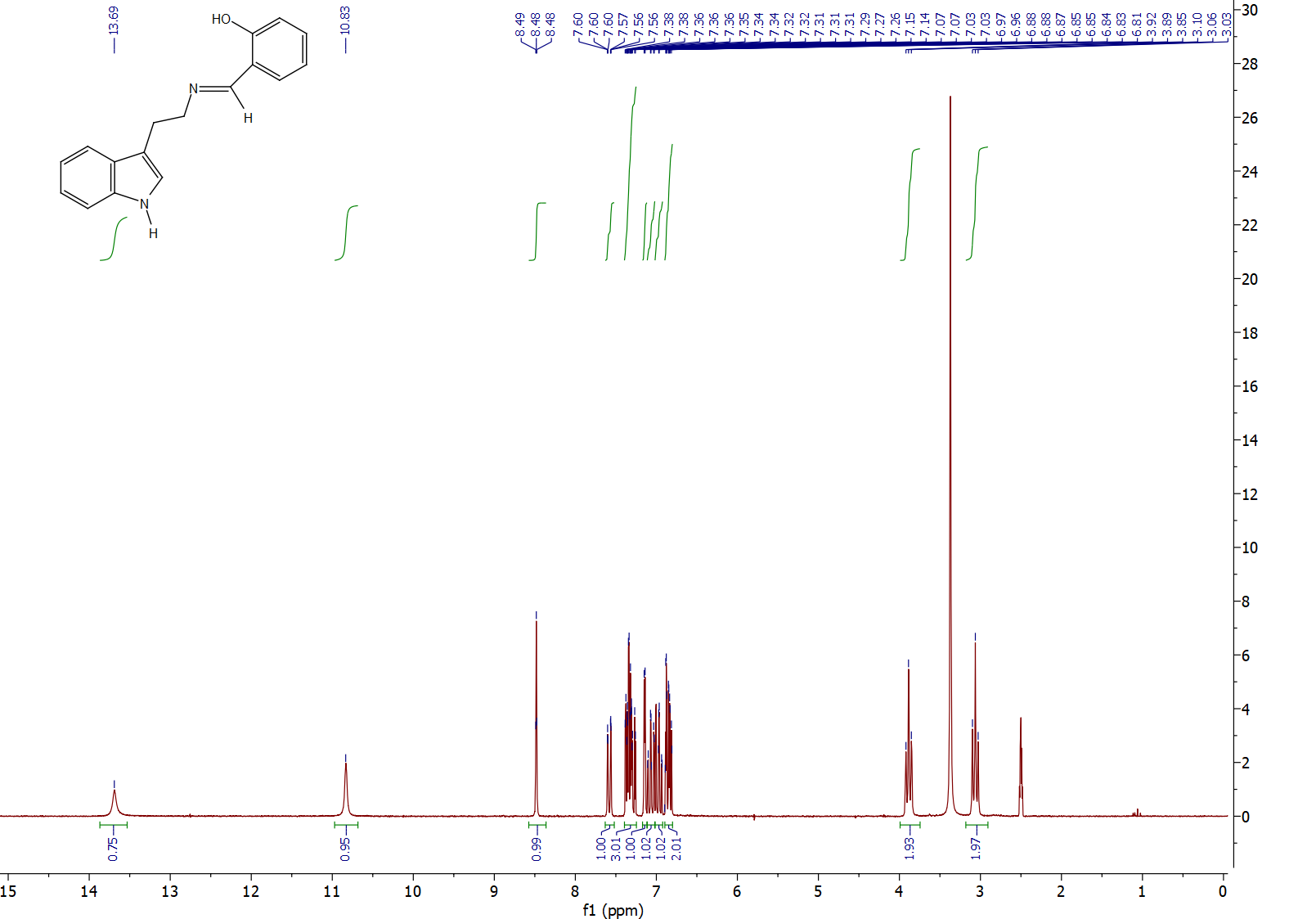


**Figure SI8**. ^13^C NMR spectra of compound **1c**


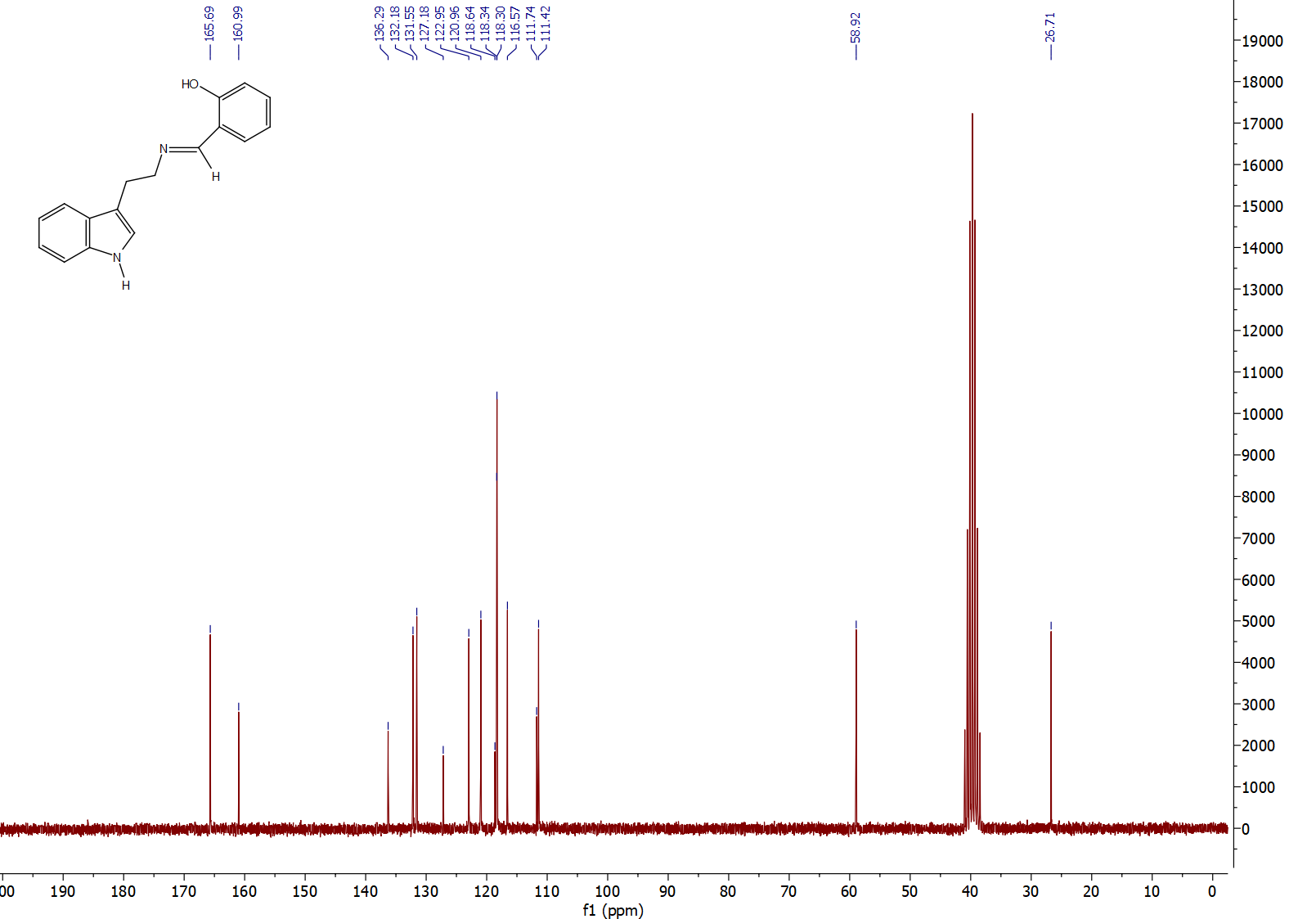


**Figure SI9.** IR spectra of compound **1c**


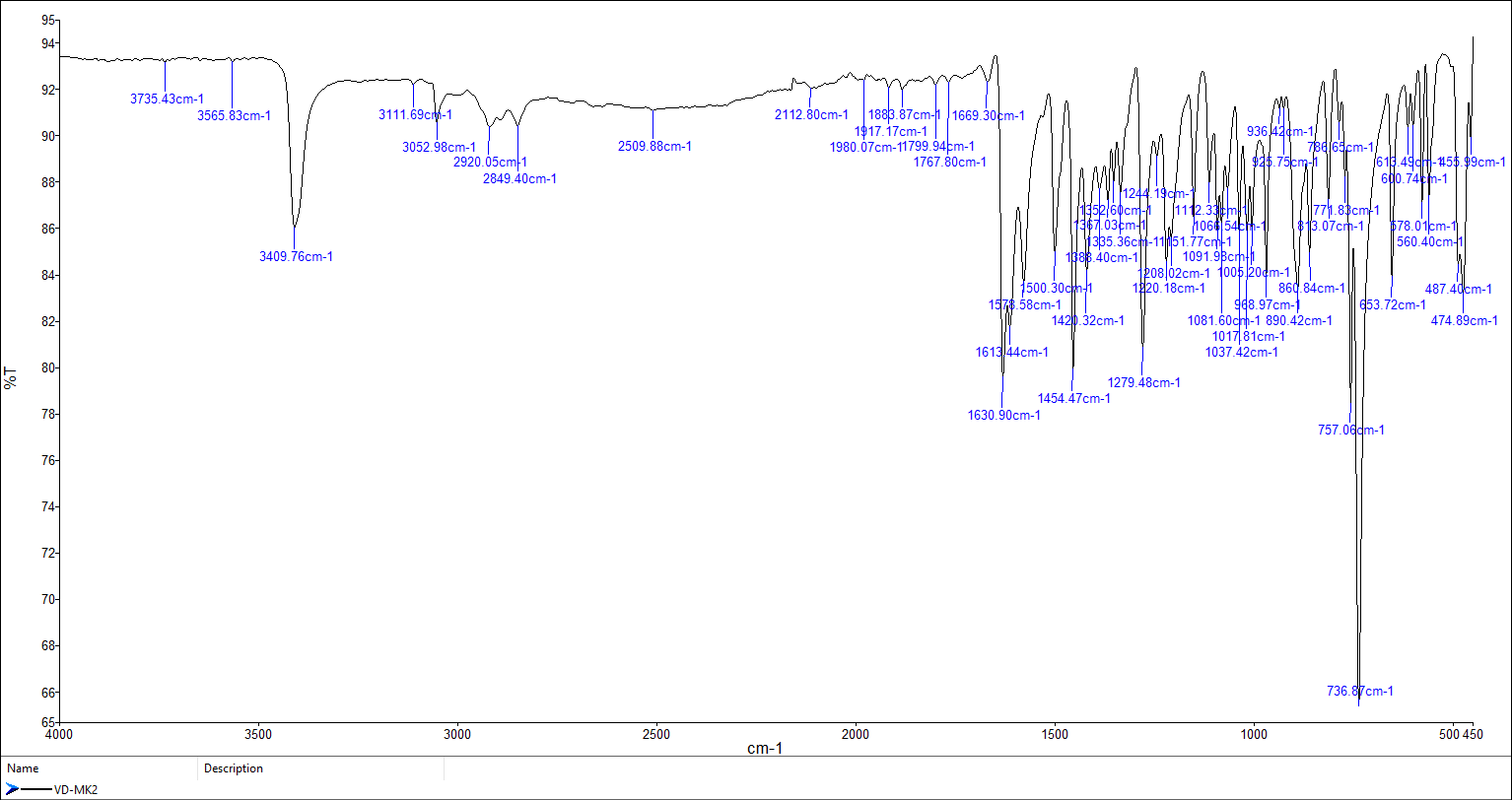


**Figure SI10.** ^1^H NMR spectra of compound **2a**


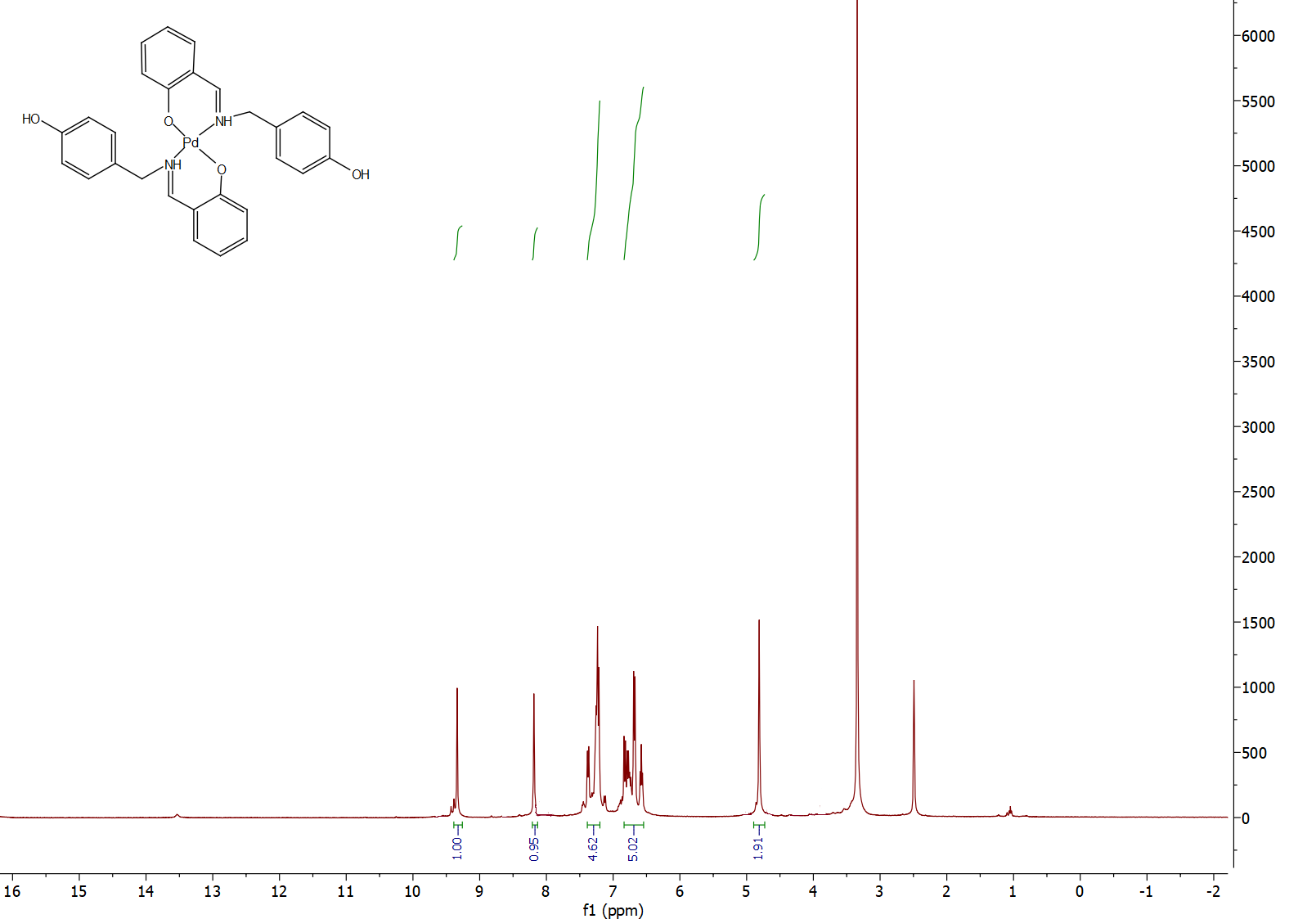


**Figure SI11.** ^13^C NMR spectra of compound **2a**


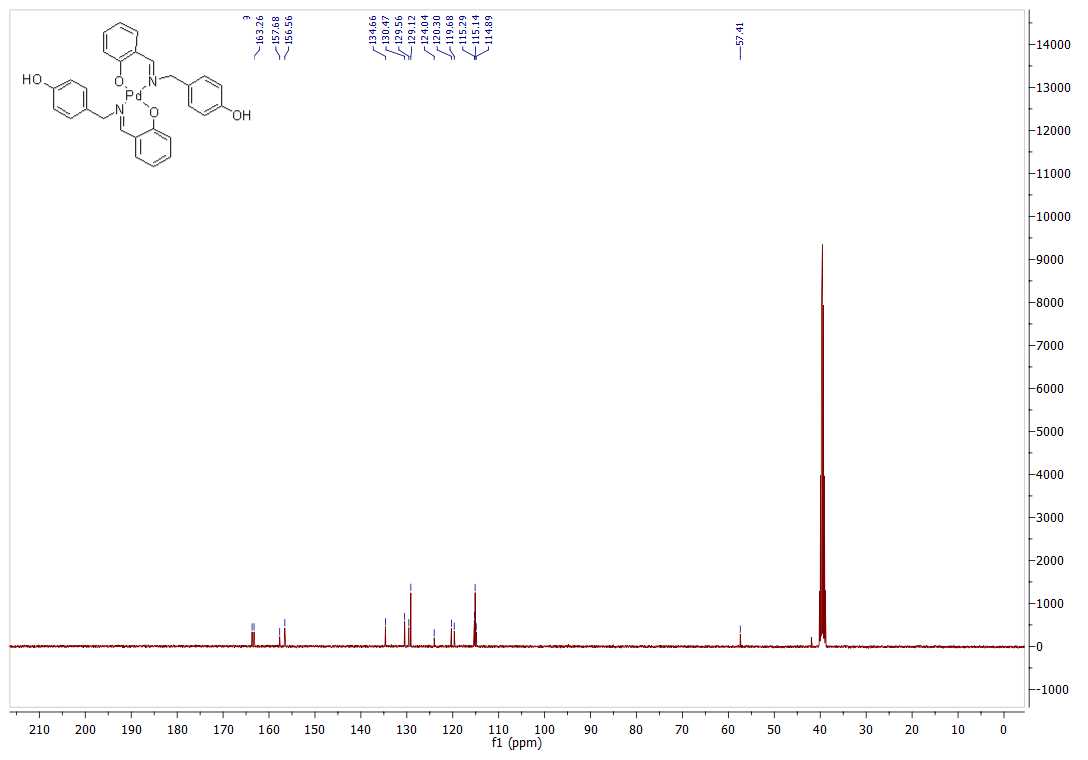


**Figure SI12.** IR spectra of compound **2a**


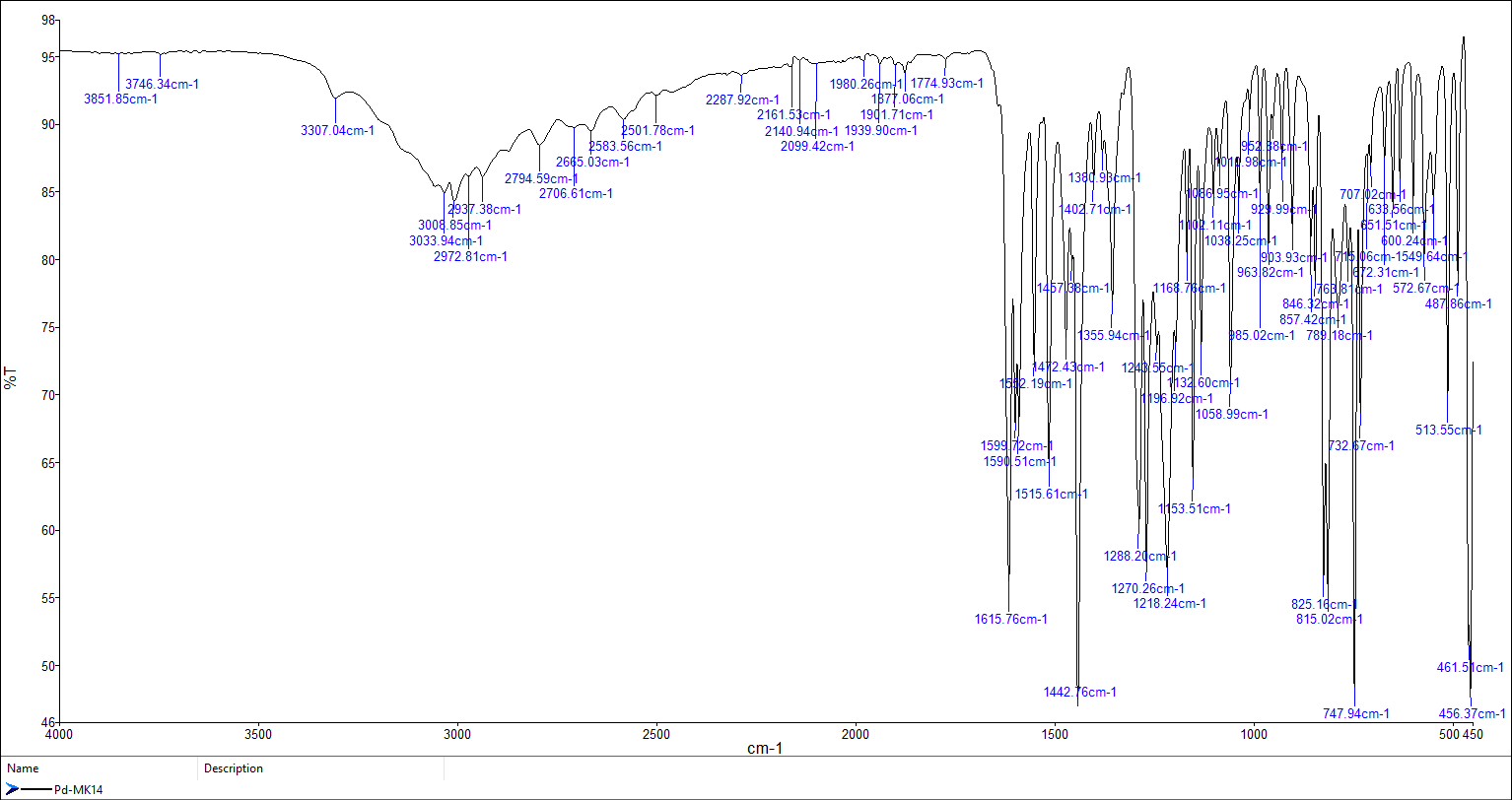


**Figure SI13**. Mass spectra of **2a**

**Figure SI14.** ^1^H NMR spectra of compound **2b**


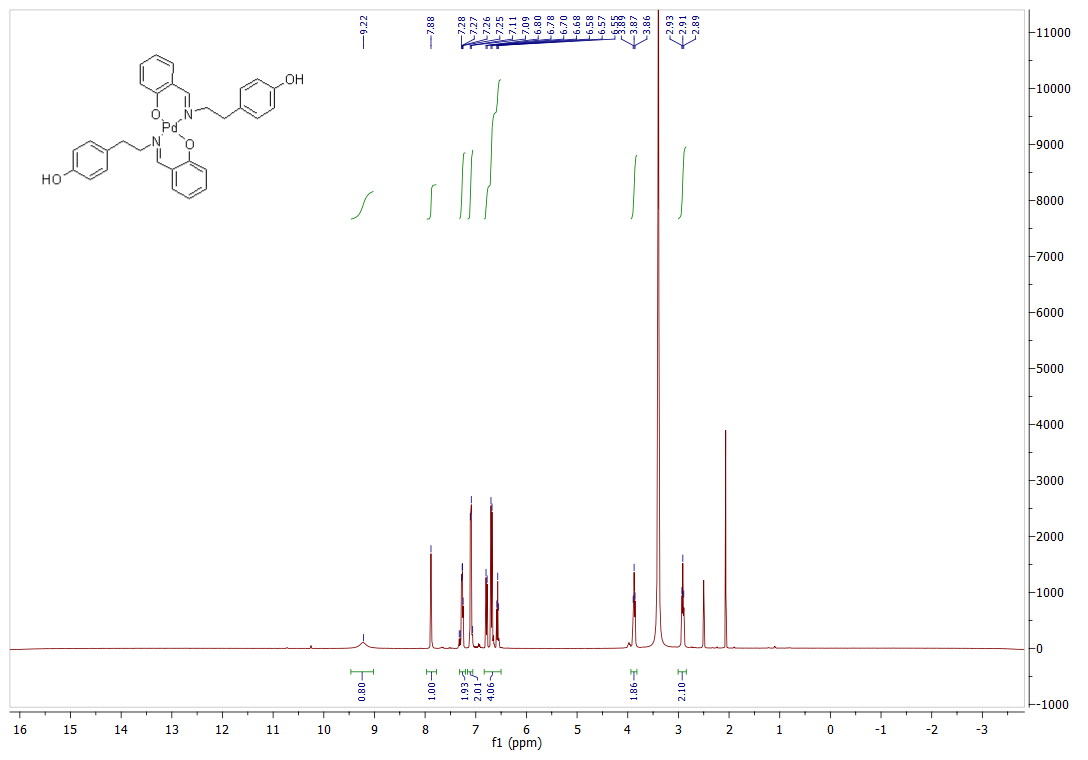


**Figure SI15.** ^13^C-NMR spectra of **2b**

**
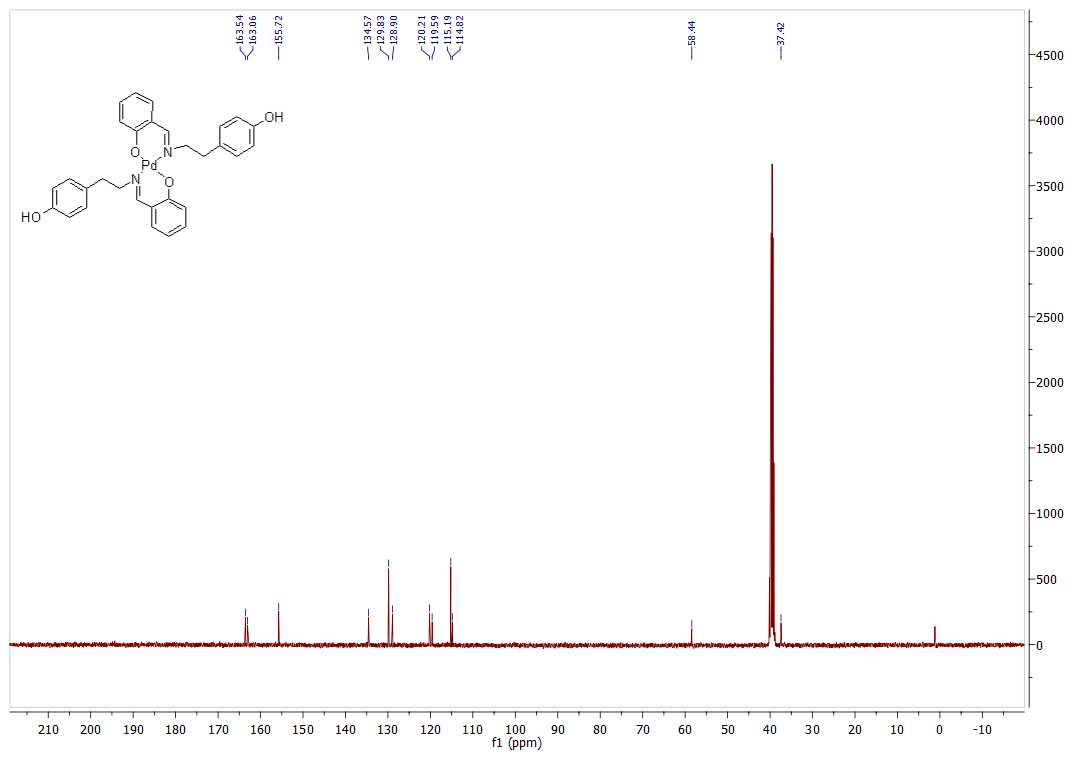
**

**Figure SI16. IR** spectra of **2b**


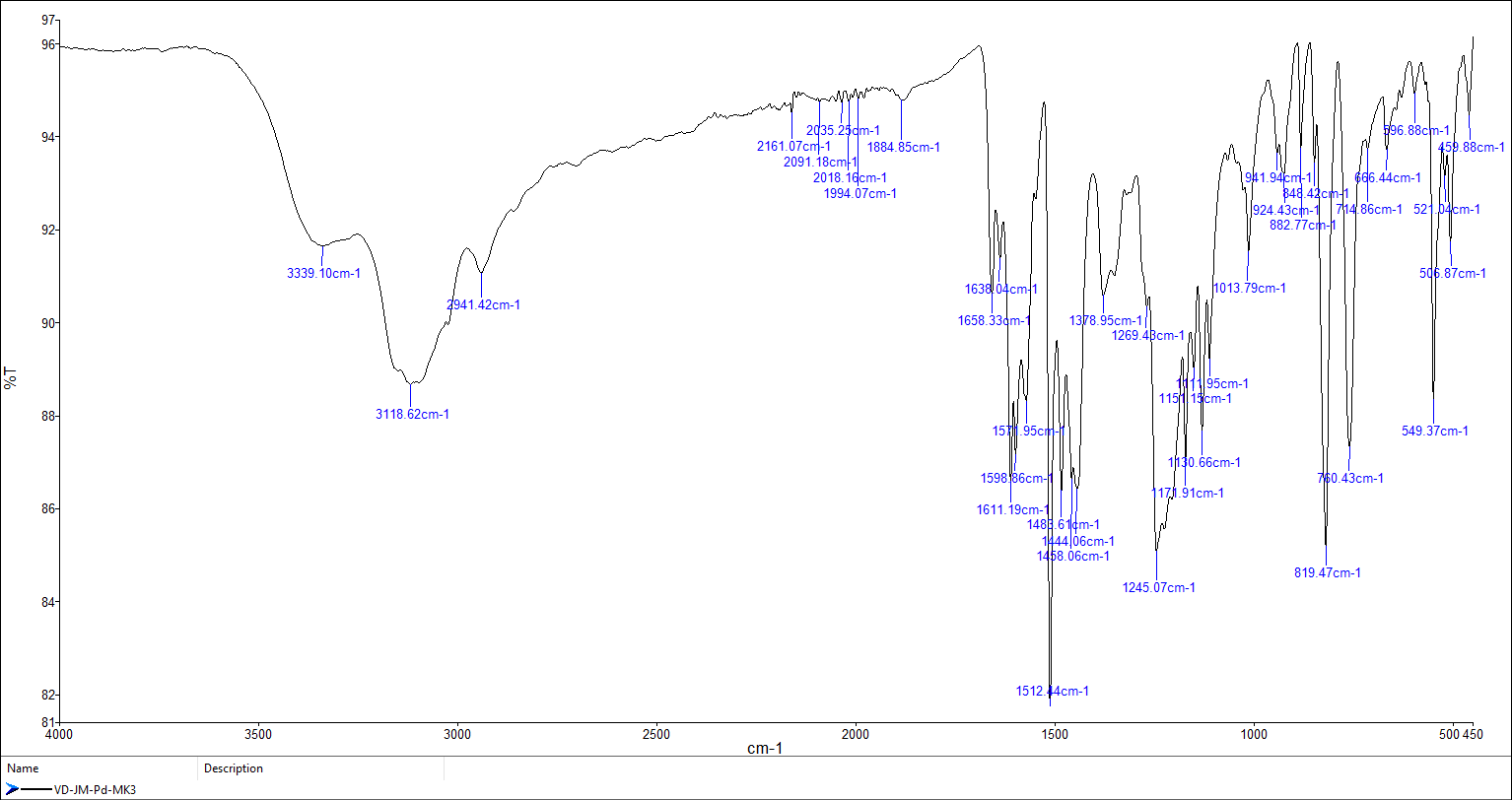


**Figure SI17**. Time-dependent UV-Vis absorption spectra for compound **2b** recorded after time t = 0, 24, 48 and 72h in DMSO as a solvent at room temperature.

**Figure SI18.** Mass spectra of compound **2b**


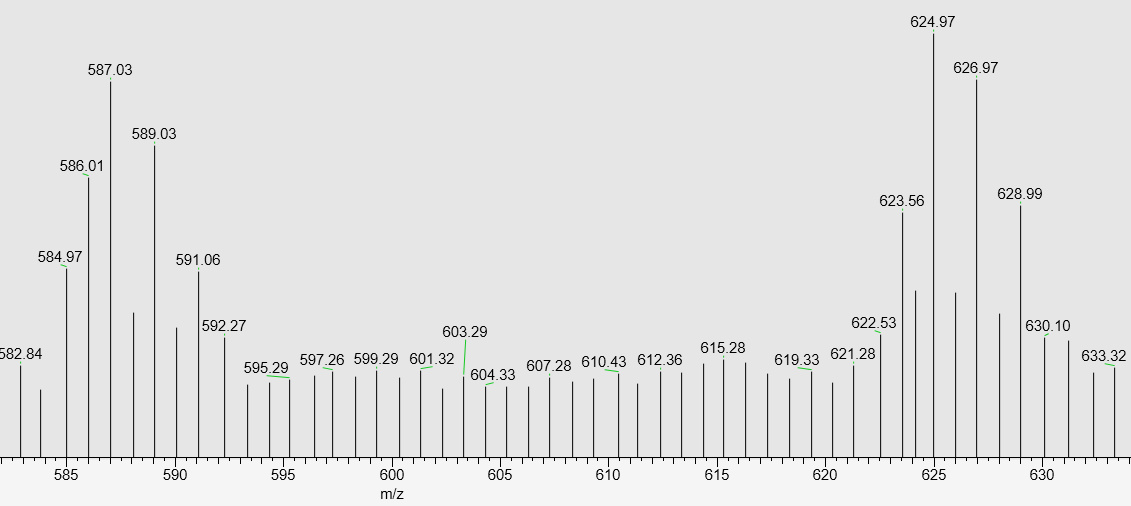


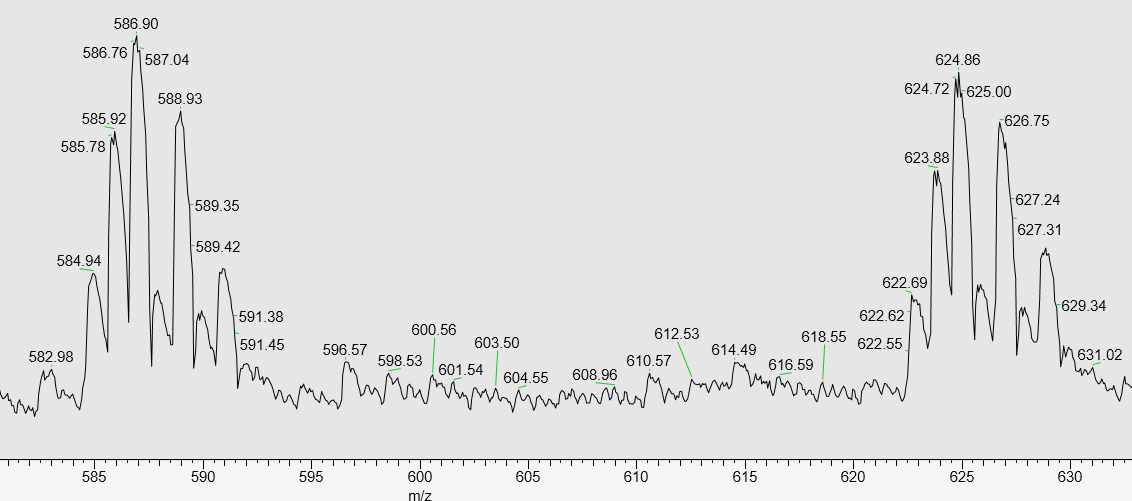


**Figure SI19.** ^1^H NMR spectra of compound **2c**


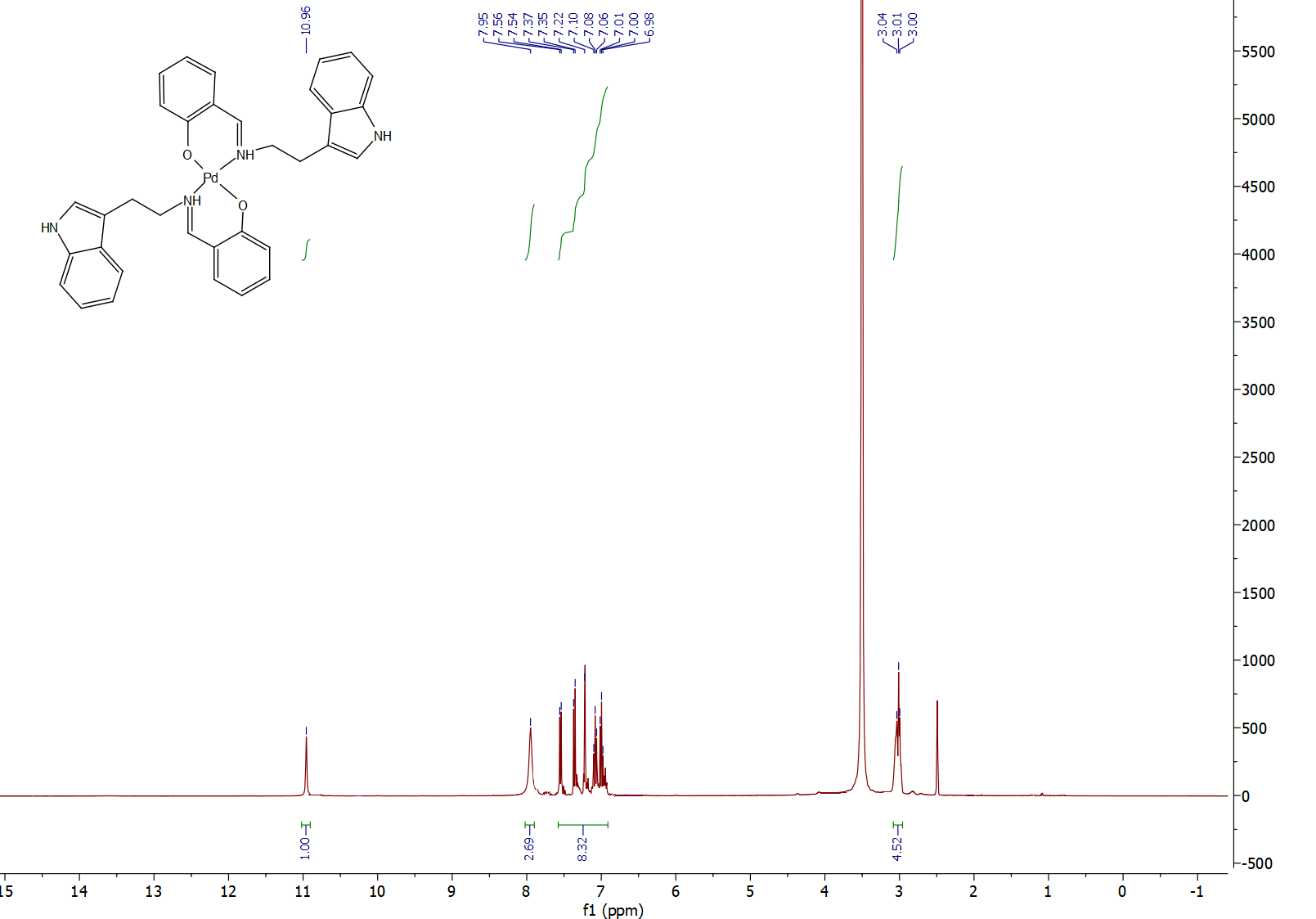


**Figure SI20.** ^13^C NMR spectra of compound **2c**


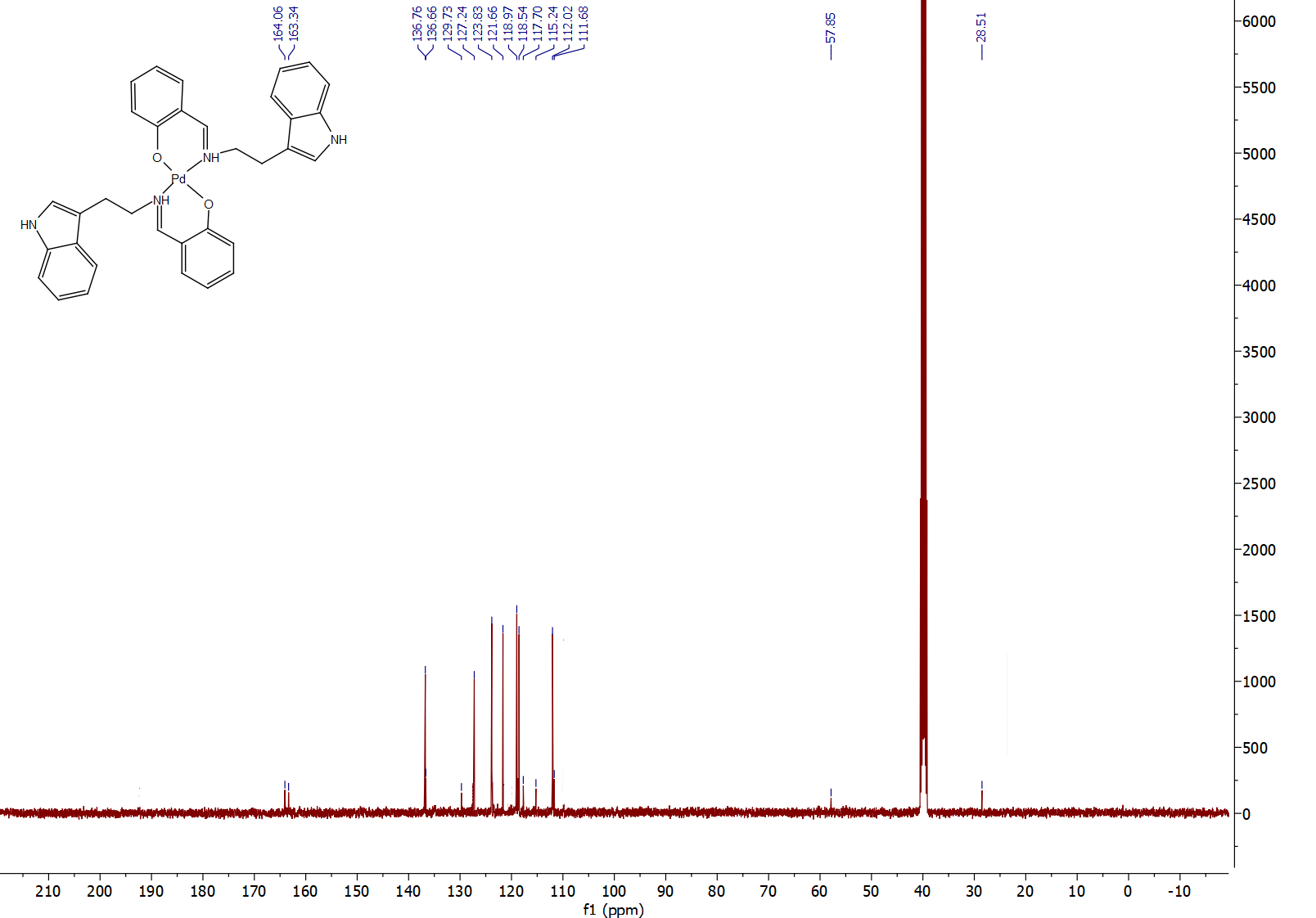


**Figure SI21.** IR spectra of **2c**


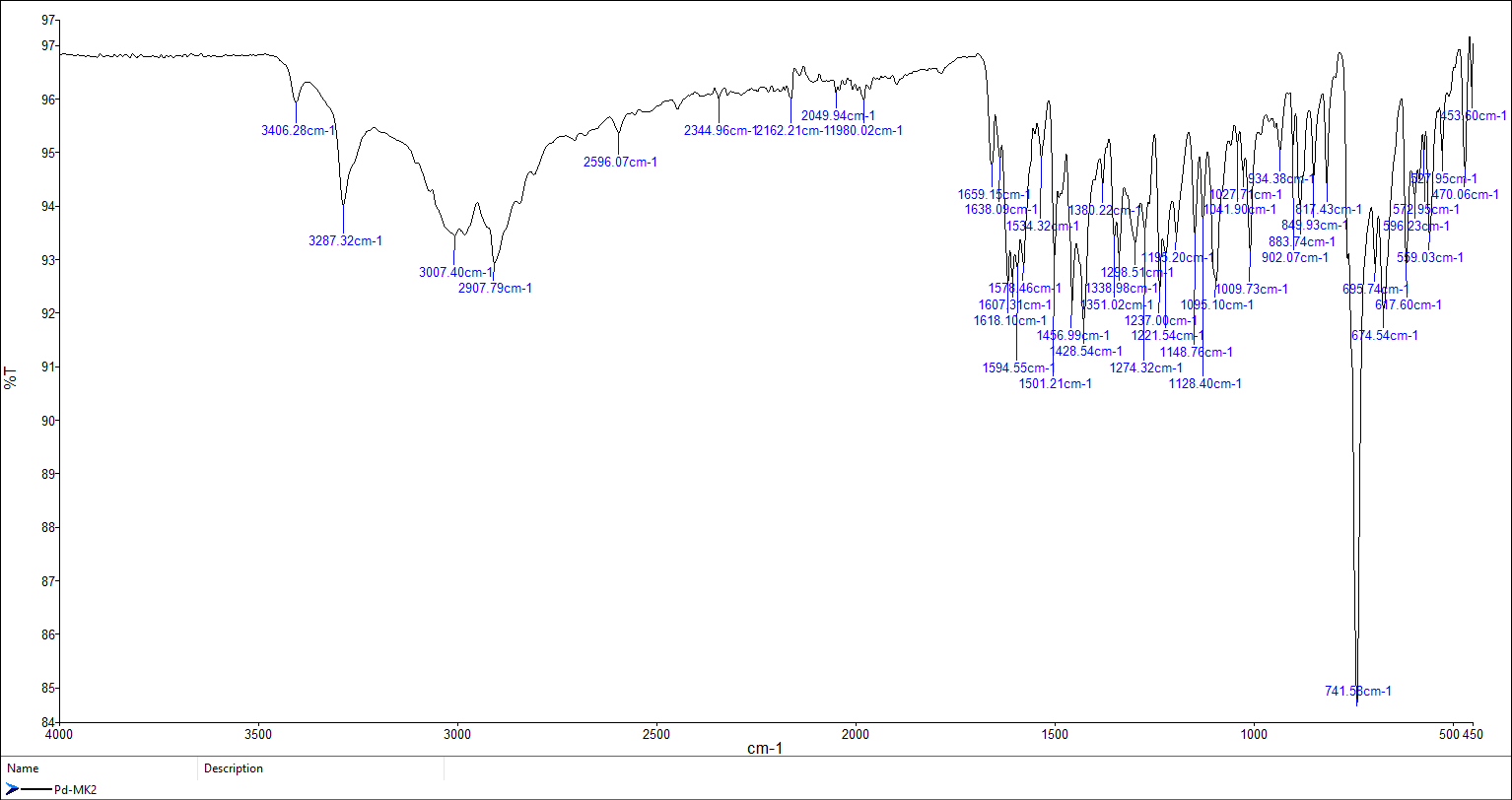


**Figure SI22.** Mass spectra of **2c**

**Figure SI23.** The stability of complexes **2b** in PBS indicated by UV-Vis spectra.

**Figure SI24.** Relative viscosity (*η/η_0_*)^1/3^ of the DNA solution in 10 mM PBS in the absence and presence of the increasing concentrations of **2c**.


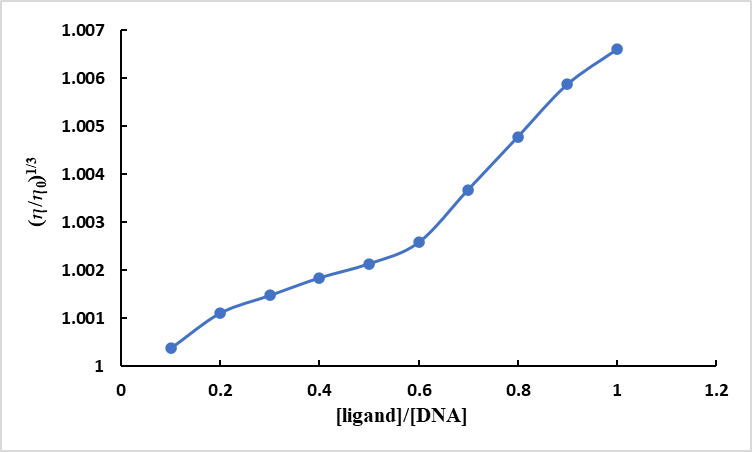


**Figure SI25.** Elemental analysis of complexes **2a-2c**


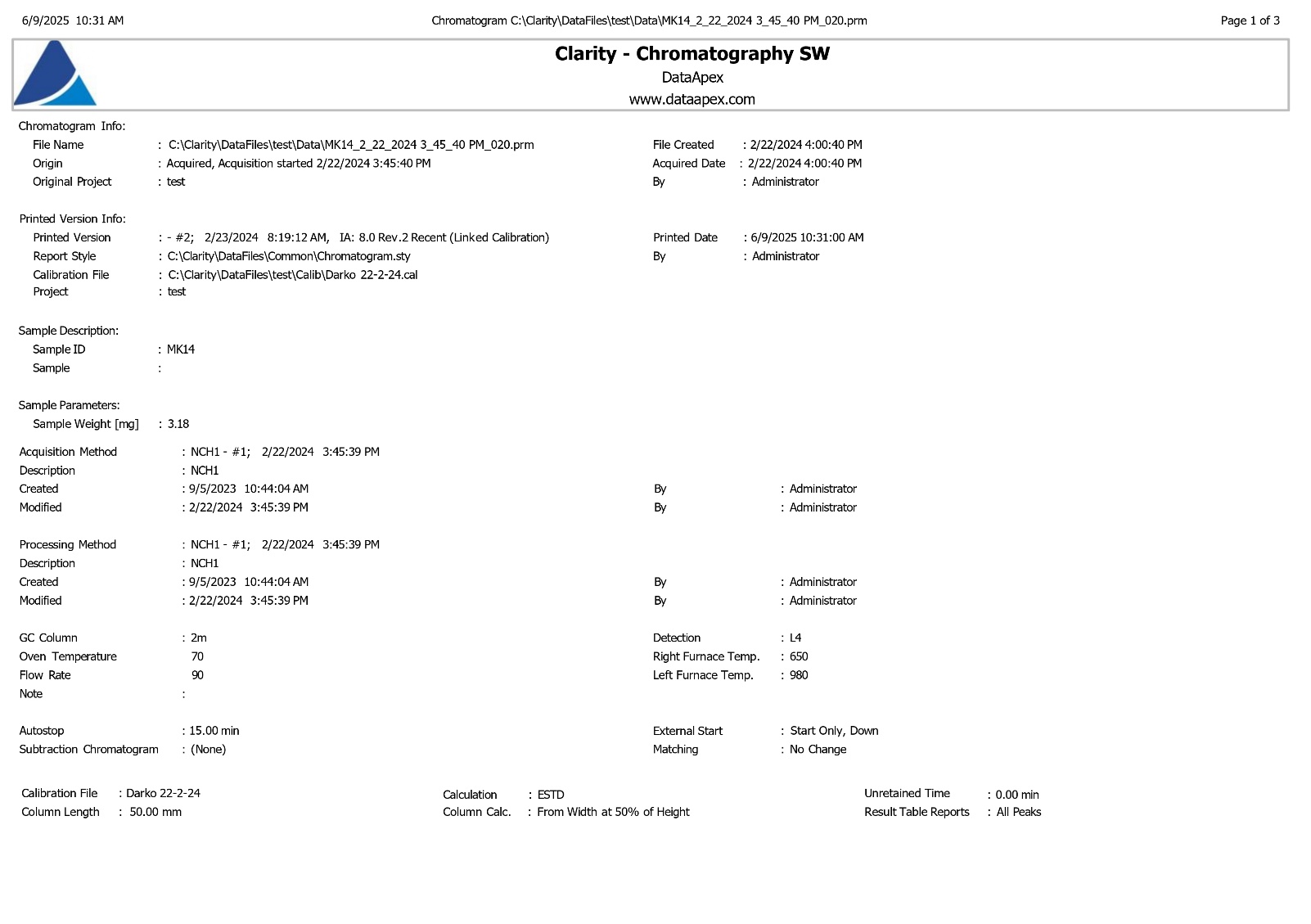


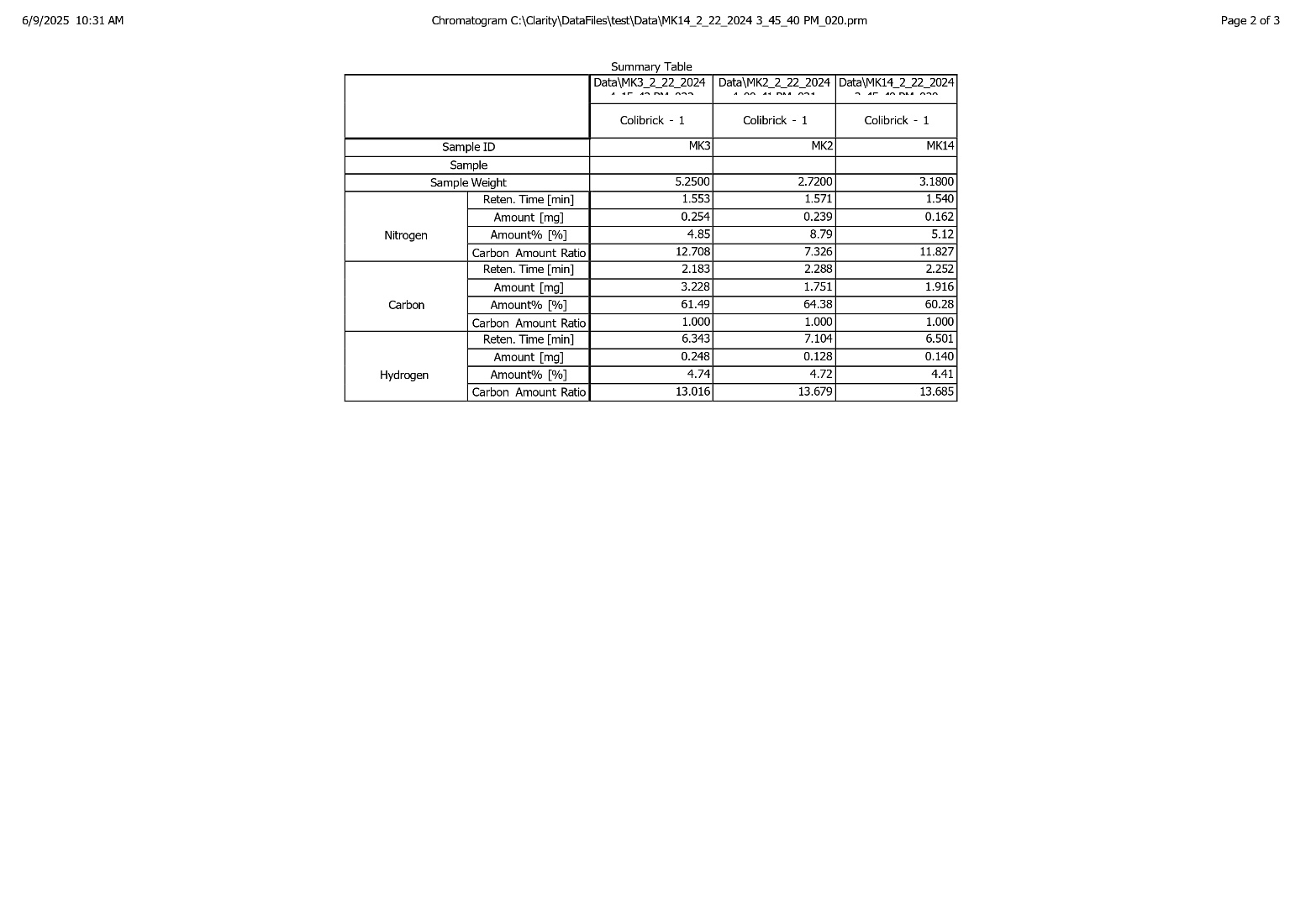


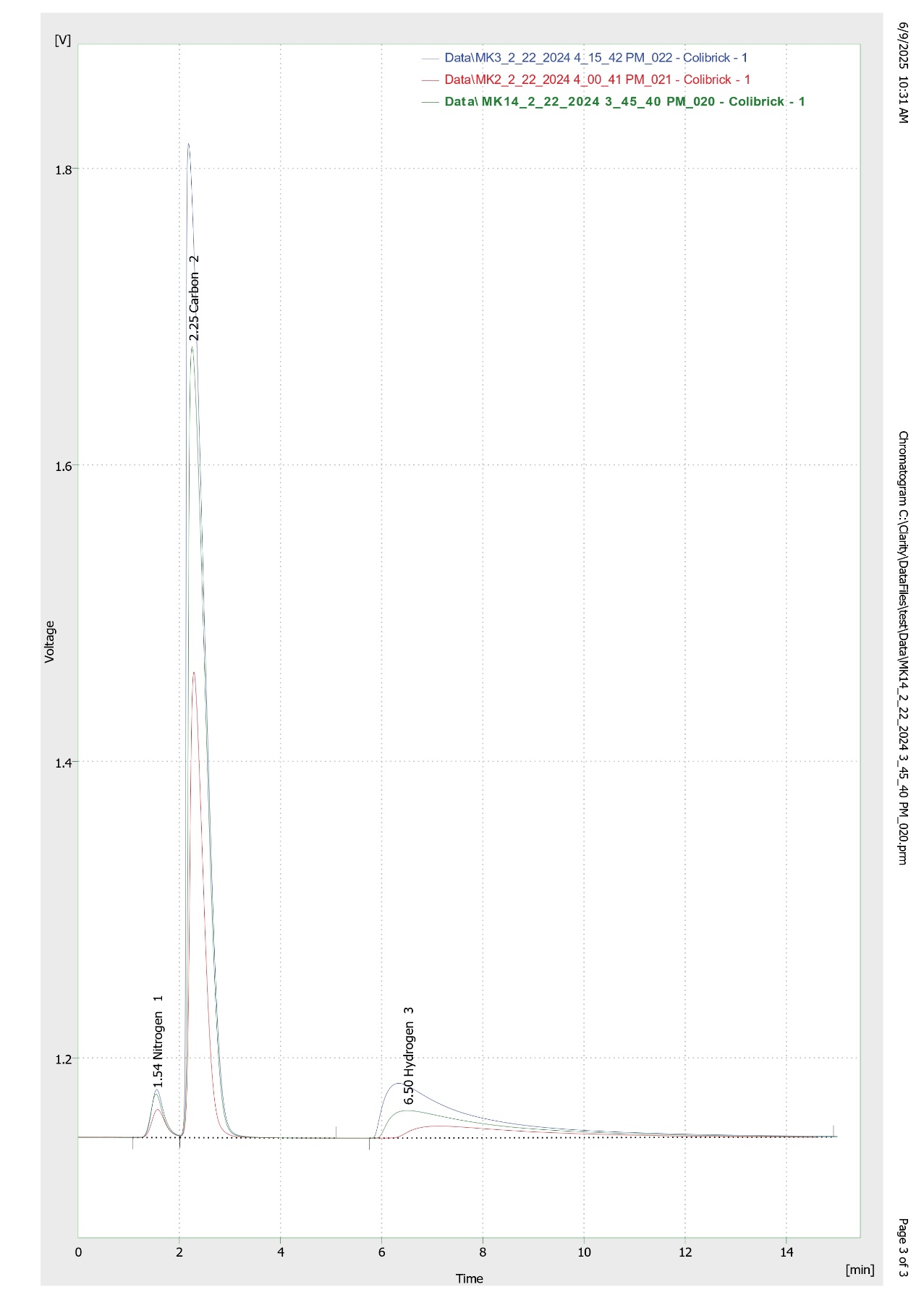

Supplement: Supplementary file 1 [file DataSheet1.docx]
